# Auditory Hyperresponsivity in Chronic Back Pain: A Randomized Controlled Trial of Pain Reprocessing Therapy

**DOI:** 10.1002/ana.78183

**Published:** 2026-02-27

**Authors:** Alina E. C. Panzel, Christian Büchel, Andrew Leroux, Tor D. Wager, Yoni K. Ashar

**Affiliations:** ^1^ Department of Systems Neuroscience University Medical Center Hamburg‐Eppendorf Hamburg Germany; ^2^ Max Planck School of Cognition Leipzig Germany; ^3^ School of Public Health University of Colorado Anschutz Medical Campus Aurora CO USA; ^4^ Department of Psychological and Brain Sciences Dartmouth College Hanover NH USA; ^5^ Division of Internal Medicine University of Colorado Anschutz Medical Campus Aurora CO USA

## Abstract

**Objective:**

Heightened sensitivity to noxious stimulation is a hallmark of chronic pain. Emerging evidence suggests heightened unpleasantness to non‐noxious (eg, auditory) aversive stimulation also characterizes chronic pain, but its magnitude, neural mechanisms, and treatment modifiability remain unknown.

**Methods:**

We compared behavioral and neural responses to auditory and pressure stimulation in 142 adults with chronic back pain (CBP) relative to 51 pain‐free controls. CBP patients then entered a randomized trial of pain reprocessing therapy (PRT) versus placebo and usual care. During functional magnetic resonance imaging, participants experienced low‐ and high‐intensity aversive sounds and mechanical pressure and provided unpleasantness ratings. Univariate analyses examined responses in primary sensory, sensory‐integrative, and midline default mode network regions. Multivariate analyses tested 4 a priori whole‐brain patterns, including patterns predictive of fibromyalgia.

**Results:**

CBP patients versus healthy controls reported heightened unpleasantness to auditory stimuli (Hedges' *g* = 0.95–1.03; *p* < 0.001) and mechanical pressure (*g* = 0.49–0.66; *p* < 0.001). For patients versus controls, auditory stimulation revealed hyperresponsivity in primary auditory cortex and insula, hyporesponsivity in the precuneus and medial prefrontal cortex (*g* = 0.33–0.59, *p* < 0.05), and increased expression of generalized and auditory‐specific aversive processing patterns (*g* = 0.33–0.39, *p* < 0.05) and of fibromyalgia‐derived multisensory sensitivity patterns (*g* = 0.43–0.50, *p* < 0.01). Longitudinal analysis found that PRT versus placebo led to reduced unpleasantness of low‐intensity auditory stimulation, along with increased medial prefrontal cortex responses for PRT versus usual care.

**Interpretation:**

CBP is associated with pronounced auditory hyperresponsivity via modality‐specific and modality‐general neural pathways, and brain mechanisms overlap with fibromyalgia. PRT versus control produced small reductions in this hyperresponsivity, suggesting potential for PRT to yield broader “central desensitization”. ANN NEUROL 20269999:n/a–n/a

Chronic back pain (CBP) affects more than 600 million people worldwide, and in most cases no peripheral cause can be identified, implicating central sensitization and other nociplastic processes.^1^, ^2^, ^3^, ^4^ Across multiple chronic pain conditions, cross‐sectional studies show patient versus control heightened unpleasantness to non‐noxious stimuli (eg, lights, odors, and sounds), which is also associated with pressure pain sensitivity and a higher likelihood of comorbid pain conditions.^5^, ^6^, ^7^, ^8^, ^9^, ^10^ Emerging longitudinal evidence suggests this multisensory reactivity is predictive of future pain: a multimodal hypersensitivity factor derived from baseline sensory testing predicted pelvic pain severity 4 years later,^11^ and in adolescents, premenarchal unpleasantness to visual and visceral stimuli predicted the development of postmenarchal widespread pain.^12^ Together, these findings suggest that chronic pain may involve broad, trait‐like sensory amplification, although causal associations are not fully understood.

Neural evidence of this non‐noxious hyperresponsivity remains limited and primarily focused on fibromyalgia, where hypoactivity of primary sensory cortices and hyperactivity in sensory‐integrative areas—especially the insula—has been observed.^13^, ^14^, ^15^ Insula hyperresponsivity to visual stimulation correlates with clinical pain intensity, and the magnitude of visual‐evoked insula responsivity reduction after treatment is associated with clinical pain relief, emphasizing the clinical relevance of non‐noxious stimulation in chronic pain.^13^ Furthermore, heightened medial prefrontal cortex (mPFC) responses to combined multisensory stimulation (visual, auditory, and tactile) has been observed in fibromyalgia,^14^ complementing findings of altered mPFC activity or connectivity across several chronic pain conditions.^16^, ^17^, ^18^, ^19^ Although its role in aversive multisensory processing remains poorly understood, the mPFC is a hub of the default mode network (DMN), involved in representing the “self in context”^20^, ^21^ and the construction of value and emotion.^22^, ^23^ Heighted mPFC responses to multisensory aversive stimuli could, therefore, reflect increased self‐relevance or increased emotional responding to aversive stimuli.

Pain reprocessing therapy (PRT) is a novel psychological treatment aiming for “central desensitization” and recovery from chronic pain. PRT led to 66% of CBP patients becoming pain‐free or nearly so post‐treatment,^24^ but whether these clinical improvements correspond to a de‐amplification of non‐noxious sensory processing remains untested.

We analyzed data from a randomized trial comparing PRT, placebo, and usual care (UC) in CBP, with a baseline cross‐sectional comparison to healthy controls. We sought to address 4 questions, focusing on behavioral and neural responses to auditory stimulation.

First, we tested whether increased subjective unpleasantness in response to non‐noxious auditory stimulation is present in an unselected community sample of CBP relative to matched controls. Studies to date have focused on fibromyalgia, and the extent of this in community CBP samples remains unclear.

Second, we investigated the neural bases of auditory hyperresponsivity, asking whether it is driven by adaptations in sensory processing pathways that are modality‐specific (eg, auditory cortex) or modality‐general (eg, insula). We compared CBP versus healthy control in region‐of‐interest (ROI) analyses and in a priori multivariate markers of modality‐specific and modality‐general aversive processing.^25^ Given the increased aversion to a range of non‐noxious stimuli in chronic pain^26^, ^27^, ^28^, ^29^ and the bidirectional relationship between chronic pain and negative affect,^5^, ^30^ we hypothesized that amplification in modality‐general aversive processing pathways drives heightened responses to non‐noxious stimulation, with potential contributions from modality‐specific pathways as well.

Third, we assessed whether neurobiological mechanisms of auditory hyperresponsivity are transdiagnostic across chronic pain conditions by applying previously developed whole‐brain multivariate markers of multisensory sensitivity in fibromyalgia.^14^ We hypothesized that CBP patients versus healthy controls would exhibit heightened expression of these fibromyalgia markers, consistent with current understandings of nociplastic mechanisms as underlying multiple pain conditions.^26^

Fourth, we examined whether auditory hyperresponsivity is treatment‐modifiable by analyzing longitudinal changes across PRT, placebo, and UC conditions. We hypothesized that PRT may decrease behavioral and neural responses to non‐noxious stimulation in comparison to other treatments, consistent with its goal of “central desensitization.”

## Methods

### Clinical Trial Design

The data presented here were collected as part of the clinical trial (Identifier: NCT03294148) comparing PRT, open‐label placebo, and UC for CPB conducted from August 2017 to November 2018 at the University of Colorado Boulder.^24^ They have not been previously published. All participants provided written informed consent as approved by the University of Colorado institutional review board.

### Participants

Adults (age, 21–70 years old) with CBP were recruited from the community in Boulder, Colorado via referrals, social media, and electronic and print announcements. CBP participants were required to report ongoing back pain for at least half the days of the last 6 months and last‐week average pain intensity score of 4 of 10 or greater during pre‐screening (although by the time of the functional magnetic resonance imaging [fMRI] session, some participants had <4 of 10 pain). Exclusion criteria aimed to yield a sample of predominantly primary (centralized) CBP and excluded self‐reported physician diagnosis of inflammatory disorders, history of metastasizing cancer, unexplained weight loss of 20lbs or more in the past year, inability to control bladder/bowel function (potential indicator of cauda equina syndrome), and leg pain greater than back pain. We further excluded participants who would likely have difficulties following research procedures or had neurological abnormalities (see Supplementary materials).

A sample of control participants with no history of chronic pain, matched in age and gender to the CBP subjects, were also recruited through community advertisements. All participants were compensated for their participation.

### Randomization and Interventions

Participants were randomized 1:1:1 using an imbalance‐minimization algorithm balancing age, sex, baseline pain, and opioid use. PRT consisted of 1 physician telehealth session and 8 psychological sessions over 4 weeks. PRT aims to help patients to reconceptualize pain as due to non‐dangerous brain activity rather than tissue injury, applying exposure‐based and emotion‐focused techniques that may support “central desensitization” and recovery from chronic pain. Placebo participants received a subcutaneous saline injection in the context of a therapeutic medical encounter. UC participants (along with both other groups) were asked to continue their ongoing treatment. The primary clinical outcome was 1‐week average back pain intensity at post‐treatment (1‐month post‐baseline). Full trial details including randomization procedures, treatment protocols, and clinical outcomes are reported in Ashar et al.^24^

### Materials and Procedures

#### Self‐report Measures of CBP

CBP severity was measured in 2 ways. First, we measured last‐week average pain intensity using the Brief Pain Inventory Short Form (BPI‐SF; 0–10 scale). Second, in the resting fMRI run immediately before the stimulation task described below, we collected spontaneous (in‐the‐moment) back pain intensity during fMRI once per minute on a visual analog scale (VAS) ranging from 0 (no pain) to 100 (worst pain imaginable) and computed the average of these ratings.

### Neuroimaging Measures

#### MRI Acquisition and Preprocessing

Structural T1 and multiband blood oxygenation level–dependent (BOLD) functional imaging (repetition time [TR] = 460 ms, echo time [TE] = 27.2 ms) were performed using a 3 T Siemens Prisma Fit MRI scanner with standard fMRI preprocessing using fMRIPrep (Supplementary materials).

#### fMRI Tasks

During functional magnetic resonance imaging (fMRI), participants completed an acute mechanical pain and aversive sound task. Participants received a pseudo‐randomized sequence of 20 pressure pain and 20 aversive audio stimulations of 6 seconds duration, at 2 intensity levels. Following each stimulation, participants rated unpleasantness on a 0 to 100 VAS. Pressure pain stimuli were administered using a device pushing a plastic piston into the left thumbnail (low intensity = 4kg/cm^2^; high = 7kg/cm^2^), manufactured by the University of Colorado Boulder CIRES Instrument Development Facility. The aversive sound (knife moving across glass) was taken from a standardized database of unpleasant sounds used and delivered at 2 fixed intensity levels without individualized loudness calibration, to preserve inter‐individual variability (and hence group differences) in sensory responsivity. The same MRI‐compatible noise‐cancelling in‐ear earbuds were used across participants with the same computer volume settings, to ensure consistent stimulus delivery. The low‐intensity stimulus was 84dB sound pressure level (SPL) and high‐intensity stimulus was 97dB SPL, as measured using a calibrated decibel meter fitted with an artificial ear connected to the same MRI earbuds (Fig S1).

## Analysis

### Behavioral Outcomes

To test baseline (cross‐sectional) group differences in unpleasantness ratings, we estimated a linear mixed model with group (CBP vs healthy control) as a between‐subject factor, stimulus intensity (high vs low) as a within‐subject factor, a group *x* intensity interaction term, age and gender as covariates, and participant random intercepts with restricted maximum likelihood estimation (*fitlme*, MATLAB 2021b, The MathWorks, Natick, MA). Outliers were identified and removed using *rmoutliers* and confirmed with visual inspection of data. To evaluate whether spontaneous back pain during the resting scan was related to task‐evoked unpleasantness, we computed the mean and variance of the spontaneous pain ratings and correlated these values with the mean evoked unpleasantness of the 4 stimulus conditions, in exploratory analyses in CBP participants. Pearson correlations characterized associations between clinical and brain variables within the CBP group.

### Functional MRI Analyses

First‐level models included boxcar regressors modeling the full 6‐second stimulus duration for both auditory and pressure stimuli, along with the unpleasantness rating period, convolved with the canonical hemodynamic response function. Covariates included 24 head motion parameters and spike regressors flagging outlying volumes (see Supplementary materials). Four contrast images conditions were estimated for each participant: low and high intensity auditory and pressure pain stimulation. Analyses were conducted using the CanlabCore toolbox and SPM12.

### ROI Analyses

**FIGURE 1:**
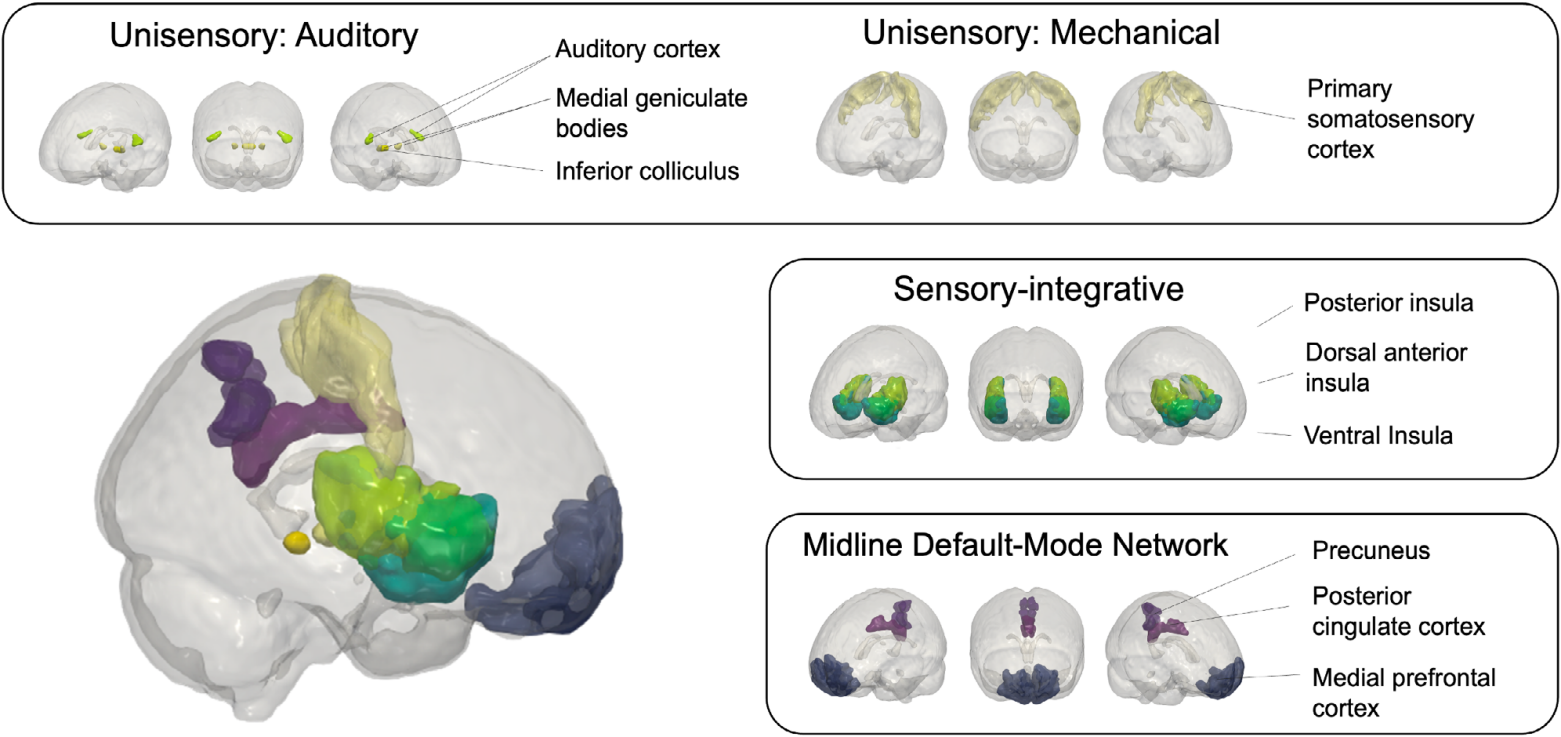
Overview of regions of interest (ROIs). ROIs were chosen to represent 3 potential neurobiological pathways of pressure and auditory hyperresponsivity in chronic back pain (CBP): unisensory, sensory‐integrative, and midline default mode network areas. [Color figure can be viewed at www.annalsofneurology.org]

To investigate the brain bases of hyperresponsivity to painful and non‐noxious stimulation we tested 3 a priori selected sets of ROIs. ROIs were selected before analysis to test different levels of the sensory processing hierarchy, and to include regions previously implicated in multisensory sensitivity in fibromyalgia^14^, ^15^ (Fig 1) and included: (1) primary sensory pathways: primary and secondary somatosensory cortex (S1, S2) in the pressure pain condition and the medial geniculate nucleus (MGN), inferior colliculus (IC), and primary auditory cortex (A1) in the auditory condition; (2) sensory integrative pathways, including 3 insula sub‐regions—the dorsal‐anterior (dIns), ventral‐anterior (vIns), and posterior insula (pIns); and (3) midline default‐mode network areas, including the mPFC, posterior cingulate cortex (PCC), and precuneus. Midline default‐mode network areas were included because of their aberrant responses to multisensory sensitivity in fibromyalgia, as well as their well‐established role in chronic pain processing.^17^, ^31^ ROIs were defined using the following atlases: Chang et al^32^ for the insula, Glasser et al^33^ for cortical areas including S1, S2, A1, mPFC PCC, and precuneus, Shen et al^34^ for the IC, and Krauth et al^35^ for MGN.

Before analysis, we identified and removed outliers within each ROI, modality, intensity, and group using *rmoutliers*. ROI average responses were extracted from contrast maps and submitted to a linear mixed model (LMM) testing for effects of group (CBP vs control) and intensity (low vs high) with random intercept per subject. For ROIs exhibiting a significant effect of group, we computed Hedges' *g* effect sizes for group differences in low and high intensity conditions separately. Primary sensory areas were tested in conditions corresponding to their expected modality (ie, MGN to sound) and in cross‐modal conditions (ie, MGN to pressure) to serve as negative controls to confirm expected unimodal responses.

#### Multivariate Pattern Analyses

Čeko and colleagues^25^ developed multivariate patterns representing generalized and sensory modality‐specific (thermal, mechanical, visual, and auditory) negative affect. We computed the cosine similarity (similar to a regularized dot product) between these patterns and our contrast images to estimate “pattern expression,” which is a measure of how strongly expressed a multivariate pattern is across the brain.

Relatedly, López‐Solà and colleagues^14^ developed 2 multivariate patterns distinguishing fibromyalgia patients from healthy controls with high accuracy (93% cross‐validated). One pattern was developed based on fMRI responses to multisensory stimulation (MSS) (combined visual, auditory, and tactile; “FM‐MSS” pattern) and 1 pattern was developed on mechanical pressure (“FM‐PAIN” pattern).^14^ We applied these patterns to our data and removed outliers and tested for group differences in pattern expression as above.

#### Classification of CBP versus Controls from Behavioral and Neural Responses

To evaluate how well hyperresponsivity to non‐noxious stimulation could distinguish CBP from controls, exploratory classifiers were developed using (1) neural data, (2) behavioral data, and (3) combined neural and behavioral data. See Supplementary Methods for analysis details.

#### Exploratory Whole‐Brain Gray Matter Cross‐Sectional Analysis

For completeness and to confirm sensibility of ROI results, we conducted whole‐brain robust regression estimating univariate (voxel‐wise) group differences in brain activity in all 4 stimulation conditions (see Supplementary materials).

#### Longitudinal Analysis of Treatment Modifiability

For auditory unpleasantness ratings, which had 20 measurements per participant, we used Gaussian location‐scale mixed models that allowed variability of ratings to vary across pre‐ and post‐intervention across participants. The model included fixed effects for group (PRT vs placebo, PRT vs UC), time (post vs pre), stimulus intensity (low vs high), time (pre‐ and post‐intervention), and their 3‐way interactions to allow for estimation of intensity specific intervention effects. The decision to adjust for baseline differences (fixed effect for group) was determined by exploratory data analysis and descriptive statistics. Additionally, the model included random effects for both the mean and variance model to account for (1) within‐person correlation induced by the study design, and (2) heterogeneity in variability of ratings across individuals. An analogous mixed effects model was used for brain measures, although simplified (no Gaussian location scaling) given there was only 4 observations per participant. The model included fixed effects for group, time, their interaction, and intensity as a covariate (therefore pooling treatment effects across intensity levels), with random intercepts and slopes per participant. Mixed models included all available data at pre‐ or post‐treatment. Robustness of findings to outliers was assessed by re‐running significant results using quantile regression (estimating the median) with the same subject‐specific random effects.

## Results

Baseline comparisons included 142 CBP subjects (52.82% female; age mean (M) [standard deviation (SD)] = 41.65 [15.65]; CBP duration = 10.25 [9.07] years) and 51 pain‐free controls (52.94% female; age = 40.65 [14.37]) (Table 1). The CBP sample was relatively high‐functioning, with little to no differences in socioeconomic status, education, or exercise habits compared to controls. In longitudinal analyses, of 50 randomized to PRT, 49 provided pre‐treatment and 43 at post‐treatment data; of 51 randomized to placebo, 49 pre‐treatment and 42 post‐treatment; and of 50 randomized to UC, 43 provided pre‐treatment and 44 post‐treatment data.

**TABLE 1:** Demographic and Pain Characteristics of CBP and Controls

Demographic and pain characteristics	CBP		Control		*p*
	n	%	n	%	
Age, mean (SD), yr	41.7	(15.6)	40.7	(14.4)	0.695
Sex					0.920
F	75	53	27	53	
M	66	47	24	47	
Education^b^					0.074
High school or less	0	0	0	0	
Some college	37	26	10	20	
College graduate	104	73	41	80	
Married	78	53	24	47	0.311
Race^a^, ^b^					0.032
American Indian or Alaskan Native	1	1	2	4	
Asian/Pacific Islander	5	4	7	14	
Black (not of Hispanic origin)	3	2	0	0	
White (not of Hispanic origin)	125	88	41	80	
Other of unknown	7	5	1	2	
Hispanic ethnicity	4	3	1	2	0.743
Employment status^b^					0.948
Full‐time (>30h/week)	81	57	28	55	
Part‐time (5–30h/week)	32	23	12	24	
Unemployed/little employed (0–5h/week)	28	20	11	21	
Subjective socioeconomic status, mean (SD)	6.7	(1.8)	6.7	(1.6)	0.831
Exercise^b^					0.070
Almost none	10	7	0	0	
1h/week	20	14	5	10	
3h/week	50	35	26	51	
7h/week	54	38	15	29	
≥14h/week	7	5	5	10	
Clinical characteristics					
BPI inference, mean (SD)	3.1	1.8	0.5	1.4	<0.001
BPI average pain, mean (SD)	3.8	1.5	0.6	1.0	<0.001
ODI, mean (SD)	22.4	10.3	2.4	4.1	<0.001
Back pain length, mean (SD)	10.2	9.1	–	–	–

Abbreviations: BPI = Brief Pain Inventory; CBP = chronic back pain; F = female; M = male; SD = standard deviation.

^a^ Race and ethnicity were collected in accord with National Institutes of Health guidelines by multiple choice self‐report.

^b^ Group differences for categorical variables were assessed using a χ^2^ test.

### Cross‐Sectional Patient Versus Control Results

#### Behavioral Results

**FIGURE 2:**
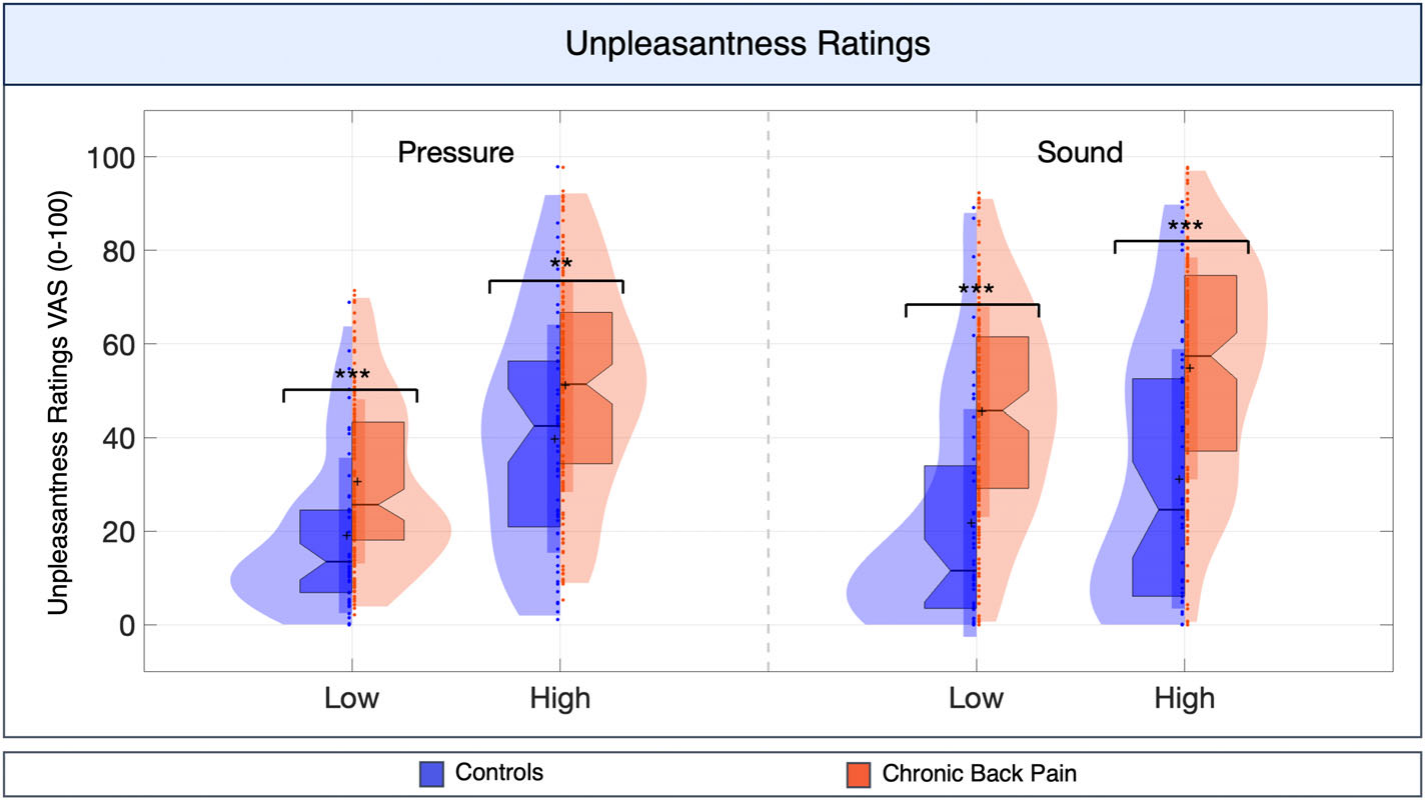
Self‐reported sensory sensitization. Subjective unpleasantness ratings in low and high intensity pressure pain and aversive sound conditions. Asterisks indicate significant group differences, **p ≤ 0.01, ***p ≤ 0.001. For detailed plot explanation see Figure S2. [Color figure can be viewed at www.annalsofneurology.org]

For both pressure and auditory conditions, CBP patients reported greater unpleasantness than controls (Fig 2) (pressure *F*_(1,45)_ = 12.30, *p* < 0.001; auditory *F*_(1,210)_ = 39.99, *p* < 0.001). There were significant effects of intensity (pressure *F*_(1,190)_ = 39.99, *p* < 0.001; auditory *F*_(1,190)_ = 37.68, *p* < 0.001), but no group × intensity interaction, indicating no group differences in intensity encoding (eg, how increasing stimulus intensity drives increased unpleasantness). Pair‐wise group comparisons showed medium to large effects, with greater unpleasantness ratings in CBP for low auditory (Hedges' *g* = 1.03), high auditory (*g* = 0.95), low pressure (*g* = 0.66), and high pressure conditions (*g* = 0.49) (group means and SDs in Table S1).

Subjective unpleasantness to auditory stimulation was associated with back pain intensity. Among CBP participants, last‐week average back pain positively correlated with auditory unpleasantness ratings at both low intensity, *r*(140) = 0.38, *p* < 0.001, and high intensity, *r*(140) = 0.30, *p* = 0.001. Exploratory analyses examining how spontaneous back pain during the resting scan was related to task‐evoked unpleasantness found that higher mean spontaneous pain was significantly associated with greater unpleasantness across all stimulus types (sound and pressure, at low and high intensities; *r* = 0.24–0.40, all *p* < 0.001) (Fig S3). Variance of spontaneous pain ratings was not significantly correlated with task‐evoked unpleasantness (all *r* < 0.07, all *p* > 0.21).

#### ROI Analyses

**FIGURE 3:**
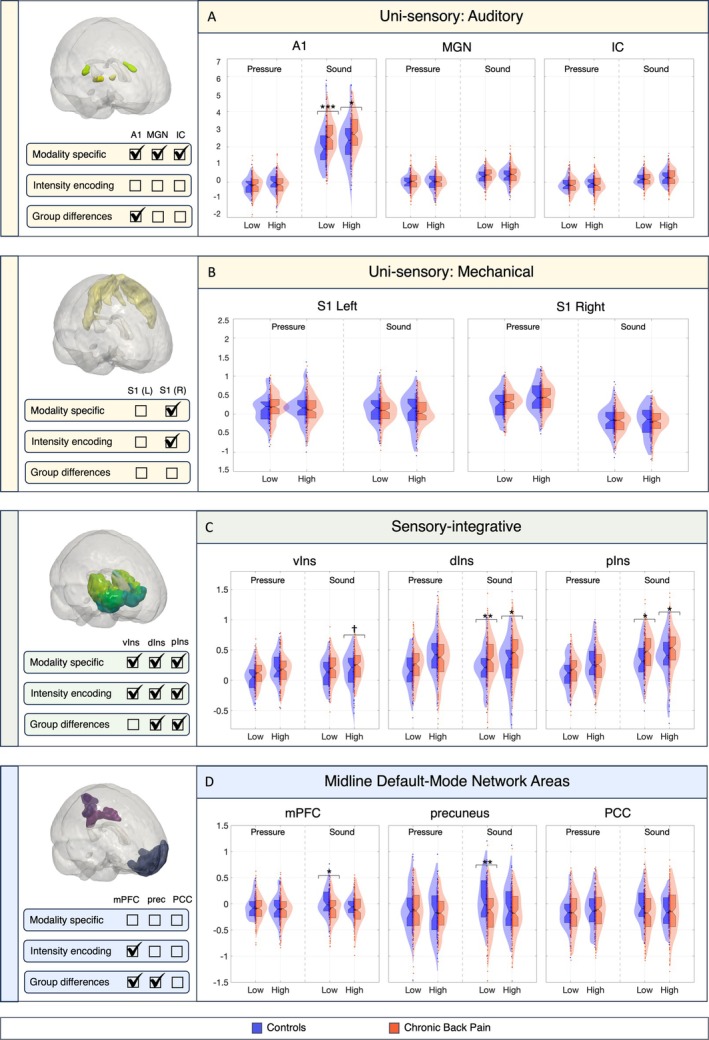
Region of interest (ROI) analysis. ROI analysis in unisensory (A,B), sensory‐integrative (C) and midline default mode network areas (D) find altered brain responses to auditory and pressure stimuli in chronic back pain versus pain‐free controls. Asterisks denote significant group differences (*p ≤ 0.05, **p ≤ 0.01, ***p ≤ 0.001). Marked checkboxes on the left indicate the results of *t* tests conducted within a ROI to assess modality specificity (low sound vs low pressure), intensity encoding (low vs high stimulus intensity within a modality), and group differences. Detailed *t* test outcomes, including means and standard deviations, are reported in Table S2.1–2.4. A1 = primary auditory cortex; dIns = dorsal‐anterior insula; IC = inferior colliculus; MGN = medial geniculate nucleus; mPFC = medial prefrontal cortex; PCC = posterior cingulate cortex; pIns = posterior insula; S1 = primary sensory cortex; vIns = ventral anterior insula. [Color figure can be viewed at www.annalsofneurology.org]

ROI analyses in primary sensory areas demonstrated modality specific responding and exhibited increased activation to high versus low intensity stimulation, as expected, and confirming ROI localization (Fig 3 and Table S2). Analyses of group differences in auditory stimulation found significantly increased A1 responses in CBP versus controls (*t*_188_ = 3.00, *p* = 0.003), with moderate to large effect sizes in low (*g* = 0.66) and high (*g* = 0.43) intensity conditions. No significant group differences were observed in either the IC or MGN. For pressure stimuli, right S1 exhibited modality‐specific activity and intensity encoding as expected, but there were no significant group differences (see Fig 3).

Sensory‐integrative ROIs revealed significant CBP versus control increases in all insula subregions during auditory, but not pressure, stimulation (see Fig 3, Table S3). The dorsal insula showed robust group differences between groups in both low (*t*_185_ = 2.94, *p* = 0.004), with medium effect sizes at low (*g* = 0.54) and high (*g* = 0.37) intensity conditions. Similarly, the posterior insula revealed greater responses in CBP compared with controls (*t*_186_ = 2.75, *p* = 0.007) with moderate effects at low (*g* = 0.43) and high (*g* = 0.36) intensity. The ventral anterior insula showed a trend‐level group difference, *t*_188_ = 1.96, *p* = 0.052, with a moderate effect size in the high‐intensity condition (*g* = 0.32).

In midline DMN areas, there were significant CBP versus control decreases in the auditory condition in the mPFC (*t*_188_ = −2.02, *p* = 0.045), with effect sizes of *g* = −0.36 to −0.20 and the precuneus (*t*_187_ = −2.77, *p* = 0.006, Hedge's *g* = −0.58 to −0.22). No significant group differences in the PCC, nor for pressure were found.

#### Multivariate Pattern Analysis of Generalized and Modality‐Specific Aversive Processing

**FIGURE 4:**
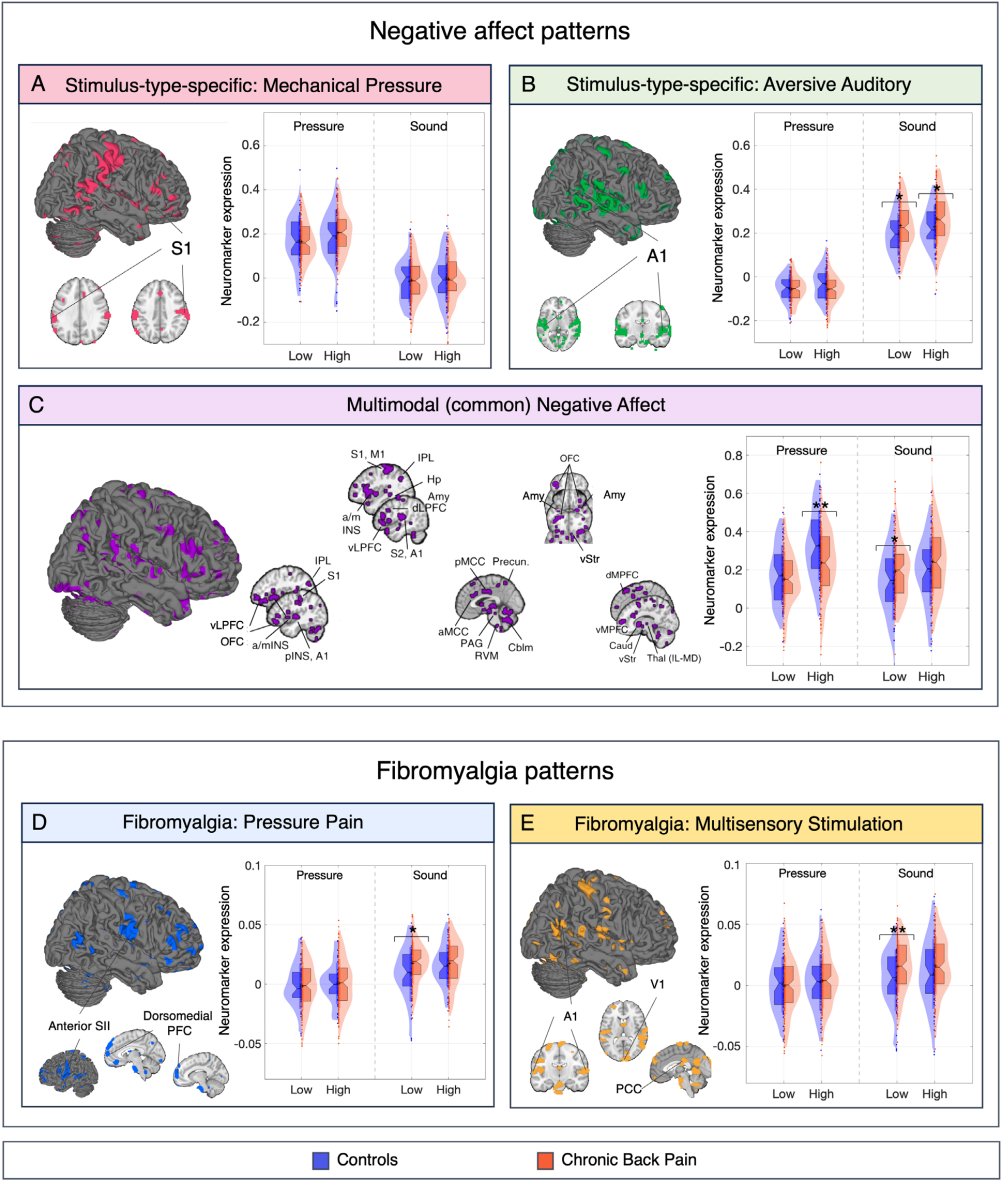
Multivariate analysis of pre‐existing negative affect patterns and fibromyalgia patterns. Multivariate pattern expression for stimulus‐type specific (mechanical pressure [A], sound [B]) and generalized (common) aversive processing (C). Multivariate pattern expression for fibromyalgia‐related pressure pain (D) and fibromyalgia‐related multisensory stimulation (E). Colored regions represent the top 10 percent of positive voxel weights. Anatomical labels indicate areas significantly contributing to predictions, as identified in original publications using different methods for estimating feature importance.^14^, ^25^ Asterisks indicate significant group differences (*p ≤ 0.05, **p ≤ 0.01, ***p ≤ 0.001). [Color figure can be viewed at www.annalsofneurology.org]

During auditory stimulation, CBP showed greater expression of both modality–sound‐specific (*t*_189_ = 2.37, *p* = 0.019) and modality‐general multivariate patterns of negative affect (*t*_186_ = 2.39, *p* = 0.018) during both low and high‐intensity auditory conditions (*g* = 0.30–0.39). During pressure stimulation, unexpectedly, there were CBP versus control decreases in the generalized negative affect marker (*t*_184_ = −2.35, *p* = 0.020) with medium effect size in the high intensity condition (*g* = −0.50) (Fig 4, Table S3).

#### Fibromyalgia Patterns

There were CBP versus control increases in pattern expression for both fibromyalgia patterns in the auditory condition (FM‐PAIN *t*_185_ = 2.22, *p* = 0.028; FM‐MSS *t*_190_ = 2.51, *p* = 0.013), with medium effect sizes *g* = 0.48 to 0.52 in the low intensity condition (see Fig 4, Table S4). For pressure stimulation, fibromyalgia pattern expression did not differ between groups.

#### Brain–Behavior Correlations

Auditory unpleasantness ratings and A1 responses were positively correlated in both CBP and in controls, for both low and high intensity sounds (*r* = 0.25–0.42). MPFC activity in the low auditory condition was positively correlated with spontaneous in‐scanner back pain ratings in CBP, *r*(140) = 0.20, *p =* 0.02. Moreover, dorsal insula activity in the low intensity auditory condition correlated positively with last‐week average back pain in CBP, *r*(140) = 0.20, *p =* 0.02. No significant correlations between clinical measures and remaining brain areas were found.

#### Behavioral and Neural Classification of CBP versus Controls

A classifier was able to separate CBP from controls with reasonable accuracy based on self‐report (area under the curve [AUC] = 0.76) and lower accuracy from neural data (AUC = 0.64). Adding neural data to behavioral data did not improve classification performance (see Supplementary Results).

#### Whole‐Brain Voxelwise Analyses

Whole‐brain gray matter voxel‐wise analysis largely confirmed results from ROI analyses, including A1 and insula hyperactivation and DMN hypoactivation in the low sound condition (Supplementary Results and Fig S4). CBP versus control deactivation was also observed in hippocampal CA1 regions during the high‐intensity sound and pressure conditions, whereas more anterior hippocampal activation was detected during the low‐intensity sound and pressure conditions (Figs S4–S8 and Table S5).

### Longitudinal Results: PRT Versus Control

#### Behavioral Results

**FIGURE 5:**
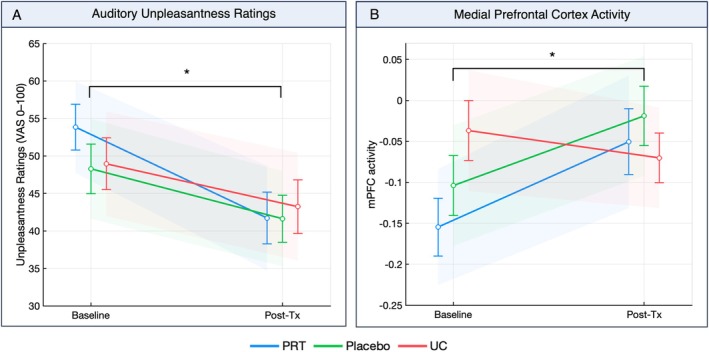
Treatment effects on auditory processing in chronic back pain. Longitudinal treatment effects on auditory unpleasantness and medial prefrontal cortex (mPFC) activity. Group‐level mean changes from baseline to post‐treatment for auditory unpleasantness ratings (A) and mPFC activity in response to auditory stimulation (B), pooled across stimulus intensity levels, for chronic back pain patients randomized to pain reprocessing therapy (PRT, blue), placebo (green), or usual care (UC, red). Shaded areas indicate 95% confidence intervals, and error bars represent standard errors of the mean. Asterisks indicate significant treatment effects across groups (*p ≤ 0.05, **p ≤ 0.01, *** *p* ≤ 0.001) [Color figure can be viewed at www.annalsofneurology.org]

PRT significantly reduced auditory unpleasantness compared to placebo for low intensity sounds (*b* = 9.72, 95% confidence interval (CI [0.79–18.66], *p* = 0.05, along with non‐significant reduction for high intensity sounds (*b* = 7.70, 95% CI [−1.22 to 16.628], *p* = 0.09) (Fig 5A, Figs S9 and S10, Table S6). Comparisons between PRT and UC showed similar trends of reduced auditory unpleasantness, which did not attain statistical significance.

#### Neural Results

Mixed‐effects modeling on brain measures revealed a significant PRT versus UC increase in mPFC responses over time, *b* = −0.14, 95% CI [−0.27 to −0.01], *p* = 0.029, estimated jointly across stimulus intensity levels (Fig 5B). These treatment‐associated increases were consistent with our findings of greater baseline deactivations for CBP versus healthy controls. No significant difference was observed for PRT versus placebo.

## Discussion

We found heightened subjective unpleasantness and neural hyper‐ and hypo‐responsivity in response to auditory stimulation in an unselected, high‐functioning community sample of CBP. Behaviorally, CBP patients rated auditory and pressure stimulation as substantially more unpleasant compared to healthy controls, with large effect sizes for auditory stimulation (*g* = 0.95–1.03) and medium effect sizes for pressure pain sensitivity (*g* = 0.49–0.66). Neurally, we found auditory hyperresponsivity in the primary auditory cortex and insula, alongside hyporesponsivity in mPFC and precuneus, indicating changes across primary sensory, sensory‐integrative, and “default mode” systems. Multivariate analyses confirmed amplification in both modality‐specific (auditory) and generalized aversive processing pathways. Fibromyalgia‐derived pattern expressions were elevated for CBP versus control, suggesting shared neural mechanisms across pain conditions. Longitudinal analysis demonstrated that PRT reduced auditory hyperresponsivity and increased mPFC activity, indicating that sensory amplification may be treatment‐modifiable.

Our results extend traditional models of pain sensitization. Although quantitative sensory testing typically focuses on somatosensory thresholds, our findings highlight that CBP involves broader amplification across sensory modalities. Group differences were more pronounced in auditory versus pressure stimulation, and neural alterations were observed predominantly in response to sound, not pressure. Moreover, higher spontaneous in‐scanner pain was linked to greater unpleasantness across all stimulus types, indicating an association between ongoing pain and cross‐modal amplification. These findings underscore the importance of aversive non‐noxious stimuli, such as auditory stimulation, in sensitization and chronic pain conditions.

The insula showed pronounced group differences in ROI analyses, with patients exhibiting hyperresponsivity to sound across posterior, dorsal anterior, and ventral subregions. Based on proposed functional specializations,^32^ posterior and dorsal anterior insula hyperactivity may reflect amplified sensory processing, whereas ventral anterior hyperactivity may reflect heightened affective appraisal, including multisensory integration, assigning emotional significance to stimuli, and interoception,^36^, ^37^, ^38^ all processes altered in chronic pain. Dorsal anterior insula responses positively correlated with patients' average clinical pain over the past week, consistent with prior observations linking this region to chronic pain intensity.^13^ Our findings build on prior research by López‐Solà et al and Harte et al,^13^, ^14^, ^15^ who both identified the insula as a key region driving multisensory reactivity in fibromyalgia. López‐Solà et al^14^, ^15^ demonstrated enhanced insular responses to multisensory stimuli was linked to clinical pain, and Harte et al^13^ found that visual‐evoked insular activity correlated with clinical pain intensity and was modulated by analgesic treatment. We extend these findings to CBP, suggesting that insula hyperresponsiveness in response to non‐noxious stimulation may be a shared neural mechanism across chronic pain conditions.

In contrast to prior work in fibromyalgia patients showing heightened DMN activity during multisensory stimulation,^14^, ^15^ we found decreased mPFC and precuneus activity in CBP versus controls in response to low intensity auditory stimulation. PRT increased mPFC responses relative to UC, suggesting partial normalization of baseline hyporesponsivity and supporting a modulatory role for PRT in sensory‐affective processing.

These findings prompted a revision of our initial hypothesis that DMN regions would show heightened responsivity in CBP, as reported in prior work in fibromyalgia.^14^ We offer 3 explanations for our mPFC findings. First, the patient versus control mPFC decreases we observed may reflect the increased subjective unpleasantness reported by patients, because the mPFC/vmPFC is typically deactivated by noxious or salient stimuli^39^, ^40^ and the magnitude of this deactivation tracks trial‐by‐trial increases in pain.^39^, ^41^ This mPFC deactivation could be explained by the increased external attentional capture of aversive stimuli, driving deactivation of the DMN broadly. A second explanation is based in findings that mPFC increases are associated with affect regulation,^42^ placebo analgesia,^43^ and endogenous pain control.^44^, ^45^ From this perspective, the greater mPFC deactivation we observed in CBP may reflect reduced engagement of regulatory processes, and PRT may thus help patients down‐regulate aversive experiences. Third, the divergence between our findings and that of Lopez‐Sola et al^14^ may be due to other factors such as differences in the stimulation paradigm‐their paradigm involved non‐aversive simultaneous multimodal stimulation whereas ours was aversive unimodal.

We also observed A1 hyperactivity in CBP, in contrast to prior evidence of auditory‐evoked A1 hypoactivity in fibromyalgia.^14^, ^15^ A1 responses correlated with auditory unpleasantness ratings in both groups, suggesting this coupling does not require priming by chronic pain. This hyperactivity may reflect the increased perceived “loudness” of sounds in CBP patients^46^ and/or increased unpleasantness or processing of the aversive content of the auditory stimulus.^47^ Notably, prior work showing A1 hypoactivity used simultaneous multisensory stimulation, as opposed to our paradigm applying each stimulus separately. Future research will be needed to investigate whether this explains different findings in A1. Last, we also observed unexpected group differences in hippocampal responses to auditory stimulation (see Supplementary Discussion).

This study further provides the first evidence that auditory hyperresponsivity in chronic pain is modifiable through psychological intervention. PRT, which aims conceptually to facilitate “central desensitization” to reduce chronic pain, also reduced auditory unpleasantness and increased mPFC responsivity. These findings imply that sensory amplification is not an immutable trait, but a dynamic and reversible process. Further research is required to understand to what extent multisensory sensitization is a cause versus consequence of chronic pain, and how treatments may jointly act on pain‐related and multisensory sensitization processes.

Limitations include the relatively small amount of within‐person data (10 trials per condition) limiting reliability^48^ and the racial differences between CBP and control, highlighting the need for more diverse samples. Treatment effects were small in magnitude, and *p* values were in the 0.01 to 0.05 range, and more robustly significant findings would increase confidence in results. Weaker pressure findings may reflect methodological variability in mechanical stimulation (anatomical differences in thumb size or nail thickness, possible thumb repositioning during scanning, variable nail bed sensitivity), whereas auditory stimuli were delivered through standardized headphones with consistent sound levels. Baseline group differences and missing data patterns could also have influenced treatment effects, although sensitivity analyses addressed potential outliers. Future studies with more trials, improved pressure paradigms, and diverse populations will strengthen generalizability.

In conclusion, auditory‐evoked increased subjective unpleasantness and neural hyper‐ and hypo‐responsivity represent a clinically relevant feature of CBP, extending beyond traditional pain sensitization. Its presence in a high‐functioning community sample suggests it may be more prevalent than previously recognized. These findings support conceptualizations of CBP in a broader context of multisensory sensitivity. Reductions in auditory hyperresponsivity with PRT indicates that responses to non‐noxious stimulation can be modified in chronic pain. Future work is needed to further untangle how pain‐related and multisensory sensitivity relate to each other, and how treatments act on 1 or both of these processes to promote health and functioning.

## Author Contributions

A.E.C.P., C.B., T.D.W., and Y.K.A. contributed to the conception and design of the study; A.E.C.P., A.L., and Y.K.A. contributed to the acquisition and analysis of data; A.E.C.P. and Y.K.A. contributed to drafting the text or preparing the figures.

## Potential Conflicts of Interest

Y.K.A. has received consulting fees from the Pain Reprocessing Therapy Center, Lin Health, and Mental Health Partners of Boulder County. Other authors declare no conflicts.

## Supporting information


**Table S1.** Unpleasantness ratings in CBP and Controls.
**Table S2.** ROI analysis: Means and Standard Deviations of CBP and Controls.
**Table S2.1.** ROI intensity encoding in CBP.
**Table S2.2.** ROI intensity encoding in Controls.
**Table S2.3.** ROI modality specificity in CBP.
**Table S2.4.** ROI modality specificity in Controls.
**Table S3.** Multivariate pattern analysis: aversive processing in CBP and Controls.
**Table S4.** Multivariate pattern analysis: Fibromyalgia pattern in CBP and Controls.
**Table S5.** Whole brain grey matter voxel wise analysis.
**Table S6.** Longitudinal Data availability.
**Figure S1.** Artificial Ear Coupler Used to Verify Auditory Stimulus Intensities. 3D printed model used with calibrated decibel meter to confirm low‐ and high‐intensity stimulus levels delivered via MRI‐compatible earbuds.
**Figure S2.** Detailed plot explanation. The plots display kernel density estimation with individual data points overlaid. Notched box plots within the violins show the median and interquartile range, while plus signs indicate the mean. The width of the violins reflects data density, and the spread illustrates response variability within each group.
**Figure S3.** Correlation heatmap of spontaneous and task‐evoked pain characteristics. Correlation heatmap showing associations between spontaneous pain characteristics (mean and variance during the resting scan) and task‐evoked unpleasantness across the four stimulus conditions (sound low, sound high, pressure low, pressure high). Bold values indicate significant correlations (*P* < .05).
**Figure S3.** Low intensity auditory stimulation. Whole brain grey‐matter analysis displaying differences in brain activity in people with CBP > controls in response to low intensity auditory stimulation. Clusters meet an exploratory threshold of *P* < 0.001 uncorrected. Yellow/orange areas display increased activity, whereas blue areas indicate hypoactivation in CBP vs controls.
**Figure S4.** High intensity auditory stimulation. Whole brain grey‐matter analysis displaying differences in brain activity in people with CBP > controls in response to high intensity auditory stimulation. Clusters meet an exploratory threshold of *P* < 0.001 uncorrected. Yellow/orange areas display increased activity, whereas blue areas indicate hypoactivation in CBP vs controls.
**Figure S5.** Low intensity pressure pain. Whole brain grey‐matter analysis displaying differences in brain activity in people with CBP > controls in response to low intensity pressure pain. Clusters meet an exploratory threshold of *P* < 0.001 uncorrected. Yellow/orange areas display increased activity, whereas blue areas indicate hypoactivation in CBP vs controls.
**Figure S6.** High intensity pressure pain. Whole brain grey‐matter analysis displaying differences in brain activity in people with CBP > controls in response to high intensity pressure pain. Clusters meet an exploratory threshold of P < 0.001 uncorrected. Yellow/orange areas display increased activity, whereas blue areas indicate hypoactivation in CBP vs controls.
**Figure S7.** Whole‐brain voxel‐wise analysis: Hippocampal Activity. Differences in hippocampal activity between CBP patients and controls, with clusters at *P* < 0.001 uncorrected. Red areas show increased activation in CBP, while blue areas indicate reduced activation. Top: Activations during low‐pressure and low‐sound conditions. Bottom: Deactivations during high‐pressure and high‐sound conditions.
**Figure S8.** Scatterplot of average reactivity across sequential exposures in the pre‐intervention assessment (x‐axis) and post‐intervention assessment (y‐axis) separately by intensity (low = red, high = blue).
**Figure S9.** Estimated sensitization effect from the primary analysis. The estimated coefficient is plotted as a solid black line, with pointwise 95% confidence intervals indicated as dashed lines.
**Figure S10.** Estimated sensitization effect from the primary analysis. The estimated coefficient is plotted as a solid black line, with pointwise 95% confidence intervals indicated as dashed lines.

## Data Availability

The data that support the findings of this study are openly available in the repository “AHinCBP” at: https://osf.io/pngkr/. MATLAB code for all analyses is available at: https://github.com/AlinaPanzel/AHinCBP.
