# Supplementary material for: Auditory Hyperresponsivity in Chronic Back Pain: A Randomized Controlled Trial of Pain Reprocessing Therapy
Source: Ann Neurol. 2026 Feb 27;99(6):1555–67. doi: 10.1002/ana.78183 (PMC13206554; doi:10.1002/ana.78183)
Supplement: Supplementary file 1 — Table S1. Unpleasantness ratings in CBP and Controls. Table S2. ROI analysis: Means and Standard Deviations of CBP and Controls. Table S2.1. ROI intensity encoding in CBP. Table S2.2. ROI intensity encoding in Controls. Table S2.3. ROI modality specificity in CBP. Table S2.4. ROI modality specificity in Controls. Table S3. Multivariate pattern analysis: aversive processing in CBP and Controls. Table S4. Multivariate pattern analysis: Fibromyalgia pattern in CBP and Controls. Table S5. Whole brain grey matter voxel wise analysis. Table S6. Longitudinal Data availability. Figure S1. Artificial Ear Coupler Used to Verify Auditory Stimulus Intensities. 3D printed model used with calibrated decibel meter to confirm low‐ and high‐intensity stimulus levels delivered via MRI‐compatible earbuds. Figure S2. Detailed plot explanation. The plots display kernel density estimation with individual data points overlaid. Notched box plots within the violins show the median and interquartile range, while plus signs indicate the mean. The width of the violins reflects data density, and the spread illustrates response variability within each group. Figure S3. Correlation heatmap of spontaneous and task‐evoked pain characteristics. Correlation heatmap showing associations between spontaneous pain characteristics (mean and variance during the resting scan) and task‐evoked unpleasantness across the four stimulus conditions (sound low, sound high, pressure low, pressure high). Bold values indicate significant correlations (P < .05). Figure S3. Low intensity auditory stimulation. Whole brain grey‐matter analysis displaying differences in brain activity in people with CBP > controls in response to low intensity auditory stimulation. Clusters meet an exploratory threshold of P < 0.001 uncorrected. Yellow/orange areas display increased activity, whereas blue areas indicate hypoactivation in CBP vs controls. Figure S4. High intensity auditory stimulation. Whole brain grey‐matter analysis displa [file ANA-99-1555-s001.docx]

Table of Content

[Methods 2](#_Toc221640369)

[1. Participants 2](#_Toc221640370)

[1.1 Exclusion Criteria. 2](#_Toc221640371)

[2. Neuroimaging Measures 2](#_Toc221640372)

[2.1 MRI Measures. 2](#_Toc221640373)

[2.2 MRI Preprocessing Pipeline. 2](#_Toc221640374)

[2.3 Denoising Pipeline. 3](#_Toc221640375)

[2.4 Classification of CBP vs. controls from behavioral and neural responses 3](#_Toc221640376)

[2.5 Exploratory Whole-Brain Gray Matter Analyses. 4](#_Toc221640377)

[2.6 Longitudinal analysis of treatment modifiability 4](#_Toc221640378)

[3. Results 6](#_Toc221640379)

[3.1 Classification results 6](#_Toc221640380)

[4. Supplementary Tables 6](#_Toc221640381)

[Table S1. Unpleasantness ratings in CBP and Controls 6](#_Toc221640382)

[Table S2. ROI analysis: Means and Standard Deviations of CBP and Controls 6](#_Toc221640383)

[Table S2.1. ROI intensity encoding in CBP 8](#_Toc221640384)

[Table S2.2. ROI intensity encoding in Controls 10](#_Toc221640385)

[Table S2.3. ROI modality specificity in CBP 11](#_Toc221640386)

[Table S2.4. ROI modality specificity in Controls 12](#_Toc221640387)

[Table S3. Multivariate pattern analysis: aversive processing in CBP and Controls 14](#_Toc221640388)

[Table S4. Multivariate pattern analysis: Fibromyalgia pattern in CBP and Controls 15](#_Toc221640389)

[Table S5. Whole brain grey matter voxel wise analysis 15](#_Toc221640390)

[Table S6. Longitudinal Data availability 19](#_Toc221640391)

[5. Supplementary Figures 20](#_Toc221640392)

[Figure S1. Detailed plot explanation. 20](#_Toc221640393)

[Figure S2. Artificial Ear Coupler Used to Verify Auditory Stimulus Intensities. 21](#_Toc221640394)

[Figure S3. Correlation heatmap of spontaneous and task-evoked pain characteristics. 22](#_Toc221640395)

[Figure S4. Low intensity auditory stimulation. 23](#_Toc221640396)

[Figure S5. High intensity auditory stimulation. 24](#_Toc221640397)

[Figure S6. Low intensity pressure pain 25](#_Toc221640398)

[Figure S7. High intensity pressure pain 26](#_Toc221640399)

[Figure S9. Scatterplot of average reactivity across sequential exposures 28](#_Toc221640400)

[Figure S10. Estimated sensitization effect from the primary analysis 28](#_Toc221640401)

[6. Discussion 28](#_Toc221640402)

[References 29](#_Toc221640403)

**Supplementary Material**

## Methods

### 1. Participants

1.1 Exclusion Criteria. Participants with self-reported schizophrenia, multiple personality disorders, dissociative identity disorder, intravenous drug usage, or a history of stroke, brain surgery, or brain tumor were excluded due to difficulties in normalizing such brains to a standard template.

### 2. Neuroimaging Measures

2.1 MRI Measures. Structural and multiband BOLD functional imaging was performed using a 3T Siemens Prisma Fit MRI scanner. Functional images were acquired using a multiband gradient-echo EPI sequence with repetition time = 460 ms, multi-band acceleration factor = 8, echo time = 27.2 ms, flip angle = 44°, number of slices = 56, slice orientation = transversal, phase encoding = © 2021 Ashar YK et al. JAMA Psychiatry. posterior to anterior, voxel size = 2.7 mm isotropic, gap between slices = 0 mm, field of view = 220 × 220 mm2, echo spacing = 0.49 ms, bandwidth = 3,048 Hz per pixel. Durations of functional scans were 8 minutes for the spontaneous pain scan, and 6 minutes for the thumb-pressure scan.

2.2 MRI Preprocessing Pipeline. Standard fMRI preprocessing procedures were used, implemented in *fMRIprep* 1.2.4 ^1^ which is based on Nipype 1.1.6. ^2^. Anatomical T1-weighted (T1w) images from both scanning sessions were corrected for intensity non-uniformity (INU) sing N4BiasFieldCorrection (ANTs 2.2.0)^3^. A T1w-reference map was computed after registration of the two T1w images (after INU-correction) using mri_robust_template ^4^. The T1w-reference was then skull-stripped using antsBrainExtraction.sh (ANTs 2.2.0), using OASIS as target template. Spatial normalization to the ICBM 152 Nonlinear Asymmetrical template version 2009c ^5^ was performed through nonlinear registration with antsRegistration, using brain-extracted versions of both the T1w volume and the template.

For the functional run, first a reference volume and its skull-stripped version were generated using a custom methodology of fMRIPrep. A deformation field to correct for susceptibility distortions was estimated based on two echo-planar imaging (EPI) references with opposing phase-encoding directions, using 3dQwarp ^6^ (AFNI 20160207). Based on the estimated susceptibility distortion, an unwarped BOLD reference was calculated for a more accurate co-registration with the anatomical reference. The BOLD reference was then co-registered to the T1w reference using flirt ^7^ (FSL 5.0.9) with the boundary-based registration cost-function ^8^. Co-registration was configured with nine degrees of freedom to account for distortions remaining in the BOLD reference. Head-motion parameters were estimated with respect to the BOLD reference before any spatiotemporal filtering using mcflirt (FSL 5.0.9).

The BOLD time-series were resampled onto their original, native space by applying a single, composite transform to correct for head-motion and susceptibility distortions. The BOLD time-series were resampled to MNI152NLin2009cAsym standard space, generating a preprocessed BOLD run in MNI152NLin2009cAsym space using antsApplyTransforms, configured with Lanczos interpolation to minimize the smoothing effects of other kernels.

2.3 Denoising Pipeline. Nuisance covariates in 1st level models included 24 head motion parameters and “spike” regressors identifying volumes with framewise displacement (FD) >= .25 mm. Spike regression was optimized for fast-TR data by a) applying a [.1 Hz – .5 Hz] band-stop filter to head motion parameters prior to computing FD, and b) computing FD with respect to the volume collected 2.4 sec previously (5 volume difference). We additionally included spike regressors for the four volumes following an identified spike, since head motion influences subsequent volumes as well.

2.4 Classification of CBP vs. controls from behavioral and neural responses**.** The neural classifier included all ROI average responses and all multivariate pattern expressions for the low sound condition, as this was the stimulation condition most distinguishing patients from controls (see Results). The behavioral classifier included unpleasantness ratings for all four conditions (low/high sound, low/high pressure). The combined classifier included both sets of features. Principal Component Analysis (PCA) was applied to reduce the dimensionality of the data prior to classification, retaining components that explained 95% of the variance. Classification performance was evaluated as the mean performance across 1000 iterations of 10-fold stratified cross-validation, to address variability induced by arbitrary selection of train/test sets, with LASSO-regularized logistic regression and Synthetic Minority Oversampling Technique ([SMOTE](https://www.mathworks.com/matlabcentral/fileexchange/75401-synthetic-minority-over-sampling-technique-smote)) to address class imbalance.

2.5 Exploratory Whole-Brain Gray Matter Analyses. Whole-brain gray matter analyses were conducted for low/high sound and pressure pain conditions ^9^. To account for unequal group sizes, inverse weights of group instance counts (1/142 for CBP, -1/51 for controls) were applied to a group dummy variable. Data were smoothed and analyzed using a two-tailed approach within a gray matter mask.

An exploratory threshold of *p* < 0.001 uncorrected with a minimum of 10 contiguous voxels was used, balancing type I and type II error rates. This threshold is often applied for hypothesis generation and meta-analyses, particularly in whole-brain studies with limited sample sizes ^10^. Neuroanatomical labeling was performed using CanlabCore tools, referencing a recent histological atlas by Amunts et al. ^11^.

2.6 Longitudinal analysis of treatment modifiability

For the statistical analysis of treatment effects on auditory unpleasantness ratings, participant- and participant/study period-specific random effects were used to account for the dependence structure induced in the data by the study design (pre-post assessments, repeated exposures within each assessment). Mixed models are robust to missing outcome data which are missing at random (MAR)^12^, and utilize all available data, including data collected from those participants who dropped out, declined to participate in one of the assessments, or had fewer than 10 usable records from a given assessment. Data visualization was used to inform model choice for both the fixed and random effects. Residuals from the final fitted model were assessed for potential residual correlation not adequately captured by the proposed fixed and random effects structures.

Models were estimated using the mgcv^13^ package in R^14^ via restricted maximum likelihood (REML)^15^. Uncertainty estimates (95% confidence intervals) were obtained using the Wald interval for (linear combinations of) regression coefficients.

Let $y_{ijk}$ denote the discomfort rating for participant $i$ during study follow-up period j at exposure k, where$i = 1,\ldots,N$; j ∈ {pre, post}; and $k = 1,\ldots,10$. Ratings were modeled using a Gaussian location–scale mixed model with nested random intercepts, nested random slopes, and a smooth exposure number effect:

Location submodel (mean):

$$\mu_{ijk} = x_{ijk}'\beta+ b_{0i} + b_{0ij} + b_{1i}\cdot k + b_{1ij}\cdot k + f₁(k)$$

Scale submodel (log-standard deviation):

$$log\left( \sigma_{ijk} \right)= z_{ijk}^{'}\gamma+ u_{0i} + u_{0ij} + u_{1i}\cdot k + u_{1ij}\cdot k$$

where:

- $x_{ijk}$: fixed-effect covariates for exposure number k, intensity (high vs low), study follow-up period (post vs pre), trial arm (PRT, placebo, usual care), and all relevant interactions (intensity × follow-up period × study arm).
- $z_{ijk}$: predictors for the scale model, including intensity, trial arm, exposure number k, and follow-up-period–specific fixed effects to allow for systematic differences in residual variability between pre- and post-intervention periods.
- $b_{0i} \sim N(0, \tau_{person, intercept}^{2})$: participant-level random intercept.
- $b_{0ij} \sim N\left( 0, \tau_{period, intercept}^{2} \right):$ follow-up-period-level random intercept nested within participant i.
- $b_{1i} \sim N(0, \tau_{expsoure, slope}^{2})$: participant-level random slope for exposure number k, allowed to correlate with b0i.
- $b_{1ij} \sim N(0, \tau_{period, slope}^{2})$:follow-up-period-specific random slope on exposure number k, independent across participants and periods.
- f₁(k) is an unspecified smooth function of exposure number k, estimated using rank-10 penalized thin-plate regression splines.
- $u_{0i} \sim N(0, \kappa_{person,intercept}^{2})$ and $u_{0ij} \sim N(0, \kappa_{period,intercept}^{2})$: nested random intercepts in the scale model.
- $u_{1i} \sim N\left( 0, \kappa_{person,slope}^{2} \right)$ and $u_{1ij} \sim N(0, \kappa_{exposure,slope}^{2})$: nested random slopes in the scale model.

RModels were fit by Restricted maximum likelihood (REML)[4]. Model fit and assumptions were assessed using residual diagnostics, quantile–quantile plots, and inspection of estimated random-effect distributions.

## 3. Results

#### 3.1 Classification results

Classification based on behavioral data achieved a mean AUC of 0.762 ± 0.009, with a sensitivity of 0.819 ± 0.041 and specificity of 0.761 ± 0.044. Classification based on neural data showed a lower AUC of 0.644 ± 0.019, with sensitivity of 0.655 ± 0.055 and specificity of 0.771 ± 0.056. The combined classifier, integrating both behavioral and neural data, performed very similarly to the classifier with behavioral data alone, with a mean AUC of 0.764 ± 0.009, sensitivity of 0.820 ± 0.042 and specificity of 0.762 ± 0.046.

### 4. Supplementary Tables

#### Table S1. Unpleasantness ratings in CBP and Controls

| **Behavioral results** | **CBP** | |  | **Control** | |  |  | **Hedge’s g** |  | ***p*-value** |  |
| --- | --- | --- | --- | --- | --- | --- | --- | --- | --- | --- | --- |
|  | mean | SD |  | mean | SD |  |  |  |  |  |  |
| Unpleasantness ratings |  |  |  |  |  |  |  |  |  |  |  |
| Sound low | 45.51 | 22.48 |  | 21.71 | 24.33 |  |  | -1.03 |  | < .001 |  |
| Sound high | 54.79 | 23.68 |  | 31.15 | 27.73 |  |  | -0.94 |  | < .001 |  |
| Pressure low | 30.6 | 17.54 |  | 19.1 | 16.61 |  |  | -0.66 |  | < .001 |  |
| Pressure high | 51.08 | 22.71 |  | 39.73 | 24.36 |  |  | -0.49 |  | .003 |  |

*Note.* Ratings on a 0 – 100 visual analog scale ranging from ‘not at all’ to ‘extremely unpleasant’.

#### Table S2. ROI analysis: Means and Standard Deviations of CBP and Controls

| **ROI Values** | **CBP** | |  | **Control** | |  | **Hedge’s g** |  | ***p*-value** |
| --- | --- | --- | --- | --- | --- | --- | --- | --- | --- |
|  | mean | SD |  | mean | SD |  |  |  |  |
| Auditory cortex |  |  |  |  |  |  |  |  |  |
| Pressure low | -0.19 | 0.48 |  | -0.20 | 0.47 |  | 0.03 |  | 0.871 |
| Pressure high | -0.18 | 0.53 |  | -0.05 | 0.52 |  | -0.25 |  | 0.126 |
| Sound low | 2.58 | 1.07 |  | 1.88 | 0.97 |  | 0.66 |  | < 0.001 |
| Sound high | 2.80 | 1.09 |  | 2.31 | 1.28 |  | 0.43 |  | < 0.05 |
| Inferior colliculus |  |  |  |  |  |  |  |  |  |
| Pressure low | -0.18 | 0.47 |  | -0.14 | 0.35 |  | -0.16 |  | 0.476 |
| Pressure high | -0.17 | 0.52 |  | -0.09 | 0.33 |  | -0.16 |  | 0.225 |
| Sound low | 0.16 | 0.40 |  | 0.18 | 0.33 |  | -0.05 |  | 0.724 |
| Sound high | 0.25 | 0.49 |  | 0.28 | 0.44 |  | -0.06 |  | 0.7 |
| Medial geniculate bodies |  |  |  |  |  |  |  |  |  |
| Pressure low | 0.03 | 0.47 |  | 0.02 | 0.37 |  | 0.03 |  | 0.833 |
| Pressure high | 0.02 | 0.46 |  | -0.01 | 0.46 |  | 0.07 |  | 0.686 |
| Sound low | 0.39 | 0.43 |  | 0.33 | 0.37 |  | 0.13 |  | 0.382 |
| Sound high | 0.45 | 0.49 |  | 0.35 | 0.42 |  | 0.20 |  | 0.21 |
| Somatosensory cortex (L) |  |  |  |  |  |  |  |  |  |
| Pressure low | 0.19 | 0.34 |  | 0.13 | 0.39 |  | 0.17 |  | 0.332 |
| Pressure high | 0.12 | 0.37 |  | 0.16 | 0.36 |  | -0.12 |  | 0.451 |
| Sound low | 0.10 | 0.33 |  | 0.14 | 0.40 |  | -0.11 |  | 0.529 |
| Sound high | 0.08 | 0.37 |  | 0.07 | 0.48 |  | 0.02 |  | 0.934 |
| Somatosensory cortex (R) |  |  |  |  |  |  |  |  |  |
| Pressure low | 0.32 | 0.33 |  | 0.37 | 0.37 |  | 0.22 |  | 0.263 |
| Pressure high | 0.43 | 0.37 |  | 0.44 | 0.41 |  | 0 |  | 0.99 |
| Sound low | -0.17 | 0.33 |  | -0.17 | 0.35 |  | 0 |  | 0.986 |
| Sound high | -0.19 | 0.32 |  | -0.21 | 0.42 |  | 0.05 |  | 0.805 |
| Ventral anterior insula |  |  |  |  |  |  |  |  |  |
| Pressure low | 0.12 | 0.20 |  | 0.05 | 0.22 |  | 0.31 |  | < 0.05 |
| Pressure high | 0.17 | 0.25 |  | 0.21 | 0.25 |  | -0.19 |  | 0.432 |
| Sound low | 0.20 | 0.25 |  | 0.13 | 0.24 |  | 0.25 |  | 0.128 |
| Sound high | 0.25 | 0.25 |  | 0.16 | 0.30 |  | 0.32 |  | 0.078 |
| Dorsal anterior insula |  |  |  |  |  |  |  |  |  |
| Pressure low | 0.25 | 0.29 |  | 0.15 | 0.30 |  | 0.34 |  | < 0.05 |
| Pressure high | 0.37 | 0.36 |  | 0.40 | 0.33 |  | -0.07 |  | 0.685 |
| Sound low | 0.36 | 0.34 |  | 0.18 | 0.30 |  | 0.54 |  | < 0.01 |
| Sound high | 0.45 | 0.38 |  | 0.31 | 0.39 |  | 0.37 |  | < 0.05 |
| Posterior Insula |  |  |  |  |  |  |  |  |  |
| Pressure low | 0.17 | 0.24 |  | 0.12 | 0.26 |  | 0.22 |  | 0.202 |
| Pressure high | 0.24 | 0.29 |  | 0.30 | 0.30 |  | 0 |  | 0.203 |
| Sound low | 0.46 | 0.32 |  | 0.33 | 0.32 |  | 0.43 |  | < 0.05 |
| Sound high | 0.53 | 0.30 |  | 0.35 | 0.35 |  | 0.36 |  | 0.05 |
| Medial prefrontal cortex |  |  |  |  |  |  |  |  |  |
| Pressure low | -0.09 | 0.23 |  | -0.05 | 0.21 |  | -0.02 |  | 0.312 |
| Pressure high | -0.1 | 0.26 |  | -0.09 | 0.26 |  | -0.02 |  | 0.901 |
| Sound low | -0.08 | 0.26 |  | 0.01 | 0.23 |  | -0.36 |  | < 0.05 |
| Sound high | -0.11 | 0.30 |  | -0.06 | 0.18 |  | -0.20 |  | 0.12 |
| Posterior cingulate cortex |  |  |  |  |  |  |  |  |  |
| Pressure low | -0.17 | 0.40 |  | -0.16 | 0.32 |  | 0 |  | 0.876 |
| Pressure high | -0.14 | 0.38 |  | -0.14 | 0.37 |  | 0 |  | 0.984 |
| Sound low | -0.18 | 0.41 |  | -0.06 | 0.34 |  | -0.32 |  | < 0.05 |
| Sound high | -0.13 | 0.44 |  | -0.16 | 0.32 |  | 0.06 |  | 0.654 |
| Precuneus |  |  |  |  |  |  |  |  |  |
| Pressure low | -0.11 | 0.41 |  | -0.09 | 0.42 |  | -0.06 |  | 0.722 |
| Pressure high | -0.18 | 0.35 |  | -0.17 | 0.50 |  | -0.03 |  | 0.871 |
| Sound low | -0.16 | 0.44 |  | 0.10 | 0.46 |  | -0.58 |  | < 0.01 |
| Sound high | -0.16 | 0.42 |  | -0.06 | 0.45 |  | -0.22 |  | 0.198 |

*Note*. Values represent contrast image parameter estimates (beta values) for region-average activity relative to baseline.

##### Table S2.1. ROI intensity encoding in CBP

| **Intensity Encoding** | **CBP** | |  | **Hedge’s g** |  | **p-value** |  |
| --- | --- | --- | --- | --- | --- | --- | --- |
|  | Mean low  (SD) | Mean high  (SD) |  |  |  |  |  |
| Auditory cortex |  |  |  |  |  |  |  |
| Sound low vs sound high | 2.55 (1.11) | 2.79 (1.09) |  | -0.22 |  | -0.06 |  |
| Pressure low vs pressure high | -0.19 (0.56) | -0.17 (0.63) |  | -0.02 |  | 0.837 |  |
| Inferior colliculus |  |  |  |  |  |  |  |
| Sound low vs sound high | 0.18 (0.43) | 0.23 (0.51) |  | -0.10 |  | 0.404 |  |
| Pressure low vs pressure high | -0.17 (0.48) | -0.14 (0.56) |  | -0.06 |  | 0.634 |  |
| Medial geniculate bodies |  |  |  |  |  |  |  |
| Sound low vs sound high | 0.40  (0.47) | 0.47 (0.53) |  | -0.15 |  | 0.216 |  |
| Pressure low vs pressure high | 0.05 (0.50) | 0.03 (0.49) |  | 0.04 |  | 0.707 |  |
| Somatosensory cortex (L) |  |  |  |  |  |  |  |
| Sound low vs sound high | 0.09 (0.36) | 0.07 (0.38) |  | 0.06 |  | 0.619 |  |
| Pressure low vs pressure high | 0.17 (0.36) | 0.15 (0.41) |  | 0.07 |  | 0.57 |  |
| Somatosensory cortex (R) |  |  |  |  |  |  |  |
| Sound low vs sound high | -0.18 (0.36) | -0.22 (0.36) |  | 0.11 |  | 0.353 |  |
| Pressure low vs pressure high | 0.33 (0.37) | 0.43 (0.37) |  | -0.27 |  | 0.024 |  |
| Ventral anterior insula |  |  |  |  |  |  |  |
| Sound low vs sound high | 0.20 (0.27) | 0.24 (0.26) |  | -0.18 |  | 0.136 |  |
| Pressure low vs pressure high | 0.11 (0.21) | 0.18 (0.27) |  | -0.28 |  | 0.02 |  |
| Dorsal anterior insula |  |  |  |  |  |  |  |
| Sound low vs sound high | 0.34 (0.38) | 0.44 (0.39) |  | -0.26 |  | 0.003 |  |
| Pressure low vs pressure high | 0.25 (0.30) | 0.38 (0.41) |  | -0.37 |  | 0.002 |  |
| Posterior insula |  |  |  |  |  |  |  |
| Sound low vs sound high | 0.45 (0.34) | 0.52 (0.33) |  | -0.19 |  | 0.104 |  |
| Pressure low vs pressure high | 0.17 (0.25) | 0.25 (0.32) |  | -0.29 |  | 0.013 |  |
| Medial prefrontal cortex |  |  |  |  |  |  |  |
| Sound low vs sound high | -0.09 (0.27) | -0.11 (0.30) |  | 0.08 |  | 0.514 |  |
| Pressure low vs pressure high | -0.08 (0.26) | -0.10 (0.28) |  | 0.08 |  | 0.509 |  |
| Posterior cingulate cortex |  |  |  |  |  |  |  |
| Sound low vs sound high | -0.19 (0.45) | -0.14 (0.44) |  | -0.13 |  | 0.278 |  |
| Pressure low vs pressure high | -0.16 (0.41) | -0.14 (0.38) |  | -0.06 |  | 0.609 |  |
| Precuneus |  |  |  |  |  |  |  |
| Sound low vs sound high | -0.17 (0.45) | -0.18 (0.45) |  | 0.02 |  | 0.84 |  |
| Pressure low vs pressure high | -0.11 (0.44) | -0.17 (0.43) |  | 0.13 |  | 0.28 |  |

*Note*. Values represent contrast image parameter estimates (beta values) for region-average activity for low vs. high intensity stimulation.

##### Table S2.2. ROI intensity encoding in Controls

| **Intensity Encoding** | **Controls** | |  | **Hedge’s g** |  | **p-value** |  |
| --- | --- | --- | --- | --- | --- | --- | --- |
|  | Mean low  (SD) | Mean high  (SD) |  |  |  |  |  |
| Auditory cortex |  |  |  |  |  |  |  |
| Sound low vs sound high | 1.95 (1.35) | 2.44 (2.22) |  | -0.27 |  | 0.179 |  |
| Pressure low vs pressure high | -0.20 (0.47) | -0.08 (0.57) |  | -0.22 |  | 0.255 |  |
| Inferior colliculus |  |  |  |  |  |  |  |
| Sound low vs sound high | 0.18 (0.33) | 0.19 (0.78) |  | 0 |  | 0.992 |  |
| Pressure low vs pressure high | -0.11 (0.38) | -0.05 (0.41) |  | -0.16 |  | 0.417 |  |
| Medial geniculate bodies |  |  |  |  |  |  |  |
| Sound low vs sound high | 0.33 (0.37) | 0.41 (0.65) |  | -0.16 |  | 0.424 |  |
| Pressure low vs pressure high | 0.02 (0.37) | -0.01 (0.46) |  | 0.06 |  | 0.774 |  |
| Somatosensory cortex (L) |  |  |  |  |  |  |  |
| Sound low vs sound high | 0.14 (0.40) | 0.13 (0.63) |  | 0.02 |  | 0.934 |  |
| Pressure low vs pressure high | 0.13 (0.39) | 0.16 (0.43) |  | -0.09 |  | 0.644 |  |
| Somatosensory cortex (R) |  |  |  |  |  |  |  |
| Sound low vs sound high | -0.15 (0.37) | -0.13 (0.69) |  | -0.03 |  | 0.88 |  |
| Pressure low vs pressure high | 0.26 (0.37) | 0.44 (0.41) |  | -0.45 |  | 0.02 |  |
| Ventral anterior insula |  |  |  |  |  |  |  |
| Sound low vs sound high | 0.12 (0.27) | 0.23 (0.72) |  | -0.2 |  | 0.3 |  |
| Pressure low vs pressure high | 0.05 (0.22) | 0.19 (0.28) |  | -0.56 |  | 0.005 |  |
| Dorsal anterior insula |  |  |  |  |  |  |  |
| Sound low vs sound high | 0.19 (0.39) | 0.36 (0.86) |  | -0.25 |  | 0.209 |  |
| Pressure low vs pressure high | 0.15 (0.30) | 0.39 (0.41) |  | -0.65 |  | < 0.001 |  |
| Posterior insula |  |  |  |  |  |  |  |
| Sound low vs sound high | 0.33 (0.32) | 0.45 (0.65) |  | -0.24 |  | 0.217 |  |
| Pressure low vs pressure high | 0.12 (0.26) | 0.28 (0.34) |  | -0.53 |  | 0.008 |  |
| Medial prefrontal cortex |  |  |  |  |  |  |  |
| Sound low vs sound high | 0.03 (0.27) | -0.07 (0.20) |  | 0.4 |  | 0.042 |  |
| Pressure low vs pressure high | -0.05 (0.21) | -0.09 (0.26) |  | 0.17 |  | 0.401 |  |
| Posterior cingulate cortex |  |  |  |  |  |  |  |
| Sound low vs sound high | -0.03 (0.37) | -0.11 (0.54) |  | 0.14 |  | 0.469 |  |
| Pressure low vs pressure high | -0.14 (0.36) | -0.14 (0.37) |  | -0.01 |  | 0.979 |  |
| Precuneus |  |  |  |  |  |  |  |
| Sound low vs sound high | 0.10 (0.46) | 0.02 (0.74) |  | 0.13 |  | 0.526 |  |
| Pressure low vs pressure high | -0.11 (0.46) | -0.17 (0.50) |  | 0.12 |  | 0.546 |  |

*Note*. Values represent contrast image parameter estimates (beta values) for region-average activity for low vs. high intensity stimulation.

#### Table S2.3. ROI modality specificity in CBP

| **Modality specific** | **CBP** | |  | **Hedge’s g** |  | **p-value** |  |
| --- | --- | --- | --- | --- | --- | --- | --- |
|  | mean  sound  (SD) | mean  pressure  (SD) |  |  |  |  |  |
| Auditory cortex |  |  |  |  |  |  |  |
| Sound low vs pressure low | 2.55 (1.11) | -0.19 (0.57) |  | 3.10 |  | < 0.001 |  |
| Sound high vs pressure high | 2.79 (1.09) | -0.17 (0.63) |  | 3.33 |  | <0.001 |  |
| Inferior colliculus |  |  |  |  |  |  |  |
| Sound low vs pressure low | 0.18 (0.43) | -0.17 (0.48) |  | 0.77 |  | <0.001 |  |
| Sound high vs pressure high | 0.23 (0.51) | -0.14 (0.56) |  | 0.69 |  | <0.001 |  |
| Medial geniculate bodies |  |  |  |  |  |  |  |
| Sound low vs pressure low | 0.40 (0.47) | 0.05 (0.50) |  | 0.70 |  | <0.001 |  |
| Sound high vs pressure high | 0.47 (0.53) | 0.03 (0.49) |  | 0.86 |  | <0.001 |  |
| Somatosensory cortex (L) |  |  |  |  |  |  |  |
| Sound low vs pressure low | 0.09 (0.36) | 0.17 (0.36) |  | -0.22 |  | 0.064 |  |
| Sound high vs pressure high | 0.07 (0.38) | 0.15 (0.41) |  | -0.19 |  | 0.107 |  |
| Somatosensory cortex (R) |  |  |  |  |  |  |  |
| Sound low vs pressure low | -0.18 (0.36) | 0.33 (0.37) |  | -1.40 |  | <0.001 |  |
| Sound high vs pressure high | -0.22 (0.36) | 0.43 (0.37) |  | -1.79 |  | <0.001 |  |
| Ventral anterior insula |  |  |  |  |  |  |  |
| Sound low vs pressure low | 0.20 (0.27) | 0.11 (0.21) |  | 0.34 |  | 0.004 |  |
| Sound high vs pressure high | 0.24 (0.26) | 0.18 (0.27) |  | 0.23 |  | 0.056 |  |
| Dorsal anterior insula |  |  |  |  |  |  |  |
| Sound low vs pressure low | 0.34 (0.38) | 0.25 (0.30) |  | 0.29 |  | 0.016 |  |
| Sound high vs pressure high | 0.44 (0.39) | 0.38 (0.41) |  | 0.15 |  | 0.195 |  |
| Posterior insula |  |  |  |  |  |  |  |
| Sound low vs pressure low | 0.45 (0.34) | 0.17 (0.25) |  | 0.96 |  | <0.001 |  |
| Sound high vs pressure high | 0.52 (0.33) | 0.25 (0.32) |  | 0.82 |  | <0.001 |  |
| Medial prefrontal cortex |  |  |  |  |  |  |  |
| Sound low vs pressure low | -0.09 (0.27) | -0.08 (0.26) |  | -0.06 |  | 0.639 |  |
| Sound high vs pressure high | -0.11 (0.30) | -0.10 (0.28) |  | -0.06 |  | 0.642 |  |
| Posterior cingulate cortex |  |  |  |  |  |  |  |
| Sound low vs pressure low | -0.19 (0.45) | -0.16 (0.41) |  | -0.07 |  | 0.531 |  |
| Sound high vs pressure high | -0.14 (0.44) | -0.14 (0.38) |  | 0 |  | 0.981 |  |
| Precuneus |  |  |  |  |  |  |  |
| Sound low vs pressure low | -0.17 (0.45) | -0.11 (0.44) |  | -0.13 |  | 0.262 |  |
| Sound high vs pressure high | -0.18 (0.45) | -0.17 (0.43) |  | -0.03 |  | 0.794 |  |

*Note*. Values represent contrast image parameter estimates (beta values) for region-average activity for sounds vs. pressure stimulation.

#### Table S2.4. ROI modality specificity in Controls

| **Modality specific** | **Control** | |  | **Hedge’s g** |  | ***p-*value** |  |
| --- | --- | --- | --- | --- | --- | --- | --- |
|  | mean  sound  (SD) | mean  pressure  (SD) |  |  |  |  |  |
| Auditory cortex |  |  |  |  |  |  |  |
| Sound low vs pressure low | 1.95 (1.35) | -0.20 (0.47) |  | 2.11 |  | <0.001 |  |
| Sound high vs pressure high | 2.44 (2.22) | -0.08 (0.57) |  | 1.54 |  | <0.001 |  |
| Inferior colliculus |  |  |  |  |  |  |  |
| Sound low vs pressure low | 0.18 (0.33) | -0.12 (0.38) |  | 0.84 | < | <0.001 |  |
| Sound high vs pressure high | 0.19 (0.78) | -0.05 (0.41) |  | 0.38 |  | 0.056 |  |
| Medial geniculate bodies |  |  |  |  |  |  |  |
| Sound low vs pressure low | 0.33 (0.37) | 0.02 (0.37) |  | 0.85 |  | <0.001 |  |
| Sound high vs pressure high | 0.41 (0.65) | -0.01 (0.46) |  | 0.74 |  | <0.001 |  |
| Somatosensory cortex (L) |  |  |  |  |  |  |  |
| Sound low vs pressure low | 0.14 (0.40) | 0.13 (0.39) |  | 0.04 |  | 0.859 |  |
| Sound high vs pressure high | 0.13 (0.63) | 0.16 (0.43) |  | -0.06 |  | 0.763 |  |
| Somatosensory cortex (R) |  |  |  |  |  |  |  |
| Sound low vs pressure low | -0.15 (0.37) | 0.26 (0.37) |  | -1.07 |  | <0.001 |  |
| Sound high vs pressure high | -0.13 (0.69) | 0.44 (0.41) |  | -0.99 |  | <0.001 |  |
| Ventral anterior insula |  |  |  |  |  |  |  |
| Sound low vs pressure low | 0.12 (0.27) | 0.05 (0.22) |  | 0.28 |  | 0.154 |  |
| Sound high vs pressure high | 0.23 (0.72) | 0.19 (0.28) |  | 0.07 |  | 0.708 |  |
| Dorsal anterior insula |  |  |  |  |  |  |  |
| Sound low vs pressure low | 0.19 (0.39) | 0.15 (0.30) |  | 0.12 |  | 0.558 |  |
| Sound high vs pressure high | 0.36 (0.86) | 0.39 (0.41) |  | -0.04 |  | 0.841 |  |
| Posterior insula |  |  |  |  |  |  |  |
| Sound low vs pressure low | 0.33 (0.32) | 0.12 (0.26) |  | 0.71 |  | <0.001 |  |
| Sound high vs pressure high | 0.45 (0.65) | 0.28 (0.34) |  | 0.33 |  | 0.095 |  |
| Medial prefrontal cortex |  |  |  |  |  |  |  |
| Sound low vs pressure low | 0.03 (0.27) | -0.05 (0.21) |  | 0.32 |  | 0.110 |  |
| Sound high vs pressure high | -0.07 (0.20) | -0.09 (0.26) |  | 0.09 |  | 0.657 |  |
| Posterior cingulate cortex |  |  |  |  |  |  |  |
| Sound low vs pressure low | -0.03 (0.37) | -0.14 (0.36) |  | 0.29 |  | 0.146 |  |
| Sound high vs pressure high | -0.10 (0.54) | -0.14 (0.37) |  | 0.08 |  | 0.688 |  |
| Precuneus |  |  |  |  |  |  |  |
| Sound low vs pressure low | 0.10 (0.46) | -0.11 (0.46) |  | 0.45 |  | 0.023 |  |
| Sound high vs pressure high | 0.02 (0.74) | -0.17 (0.50) |  | 0.30 |  | 0.131 |  |

*Note*. Values represent contrast image parameter estimates (beta values) for region-average activity for sounds vs. pressure stimulation.

#### Table S3. Multivariate pattern analysis: aversive processing in CBP and Controls

| **Multivariate patterns of negative affect** | **CBP** | |  | **Control** | |  | **Hedge’s *g*** |  | ***p-*value** |
| --- | --- | --- | --- | --- | --- | --- | --- | --- | --- |
|  | mean | SD |  | mean | SD |  |  |  |  |
| Common generalized negative affect |  |  |  |  |  |  |  |  |  |
| Pressure low | 0.15 | 0.15 |  | 0.17 | 0.16 |  | -0.14 |  | 0.393 |
| Pressure high | 0.24 | 0.18 |  | 0.33 | 0.18 |  | -0.50 |  | < 0.01 |
| Sound low | 0.19 | 0.16 |  | 0.13 | 0.19 |  | 0.37 |  | < 0.05 |
| Sound high | 0.24 | 0.18 |  | 0.19 | 0.18 |  | 0.30 |  | 0.074 |
| Stimulus-type specific: pressure pain |  |  |  |  |  |  |  |  |  |
| Pressure low | 0.16 | 0.09 |  | 0.17 | 0.11 |  | 0.04 |  | 0.816 |
| Pressure high | 0.21 | 0.10 |  | 0.18 | 0.13 |  | 0.22 |  | 0.232 |
| Sound low | -0.01 | 0.10 |  | -0.01 | 0.10 |  | 0.05 |  | 0.767 |
| Sound high | 0 | 0.11 |  | 0 | 0.11 |  | -0.04 |  | 0.796 |
| Stimulus-type specific: aversive auditory |  |  |  |  |  |  |  |  |  |
| Pressure low | -0.06 | 0.06 |  | -0.05 | 0.06 |  | 0.06 |  | 0.705 |
| Pressure high | -0.06 | 0.07 |  | -0.03 | 0.08 |  | -0.35 |  | < 0.05 |
| Sound low | 0.24 | 0.11 |  | 0.19 | 0.10 |  | 0.39 |  | < 0.05 |
| Sound high | 0.27 | 0.12 |  | 0.23 | 0.10 |  | 0.34 |  | < 0.05 |

*Note*. Values represent pattern expression, computed as the cosine similarity of the multivariate pattern and the contrast image (stimulation vs. baseline).

#### Table S4. Multivariate pattern analysis: Fibromyalgia pattern in CBP and Controls

| **Multivariate fibromyalgia patterns** | **CBP** | |  | **Control** | |  | **Hedge’s g** |  | ***p-*value** |
| --- | --- | --- | --- | --- | --- | --- | --- | --- | --- |
|  | mean | SD |  | mean | SD |  |  |  |  |
| FM Pain |  |  |  |  |  |  |  |  |  |
| Pressure low | 0 | 0.02 |  | 0 | 0.02 |  | 0.01 |  | 0.971 |
| Pressure high | 0 | 0.02 |  | 0 | 0.02 |  | 0.01 |  | 0.968 |
| Sound low | 0.02 | 0.02 |  | 0.01 | 0.02 |  | 0.48 |  | < 0.05 |
| Sound high | 0.02 | 0.02 |  | 0.02 | 0.02 |  | 0.09 |  | 0.571 |
| FM Multisensory |  |  |  |  |  |  |  |  |  |
| Pressure low | 0 | 0.02 |  | 0 | 0.02 |  | 0.04 |  | 0.81 |
| Pressure high | 0 | 0.02 |  | 0 | 0.02 |  | 0.06 |  | 0.724 |
| Sound low | 0.02 | 0.02 |  | 0 | 0.03 |  | 0.52 |  | < 0.01 |
| Sound high | 0.02 | 0.02 |  | 0.01 | 0.03 |  | 0.26 |  | 0.146 |

*Note*. Values represent pattern expression, computed as the cosine similarity of the multivariate pattern and the contrast image (stimulation vs. baseline).

#### Table S5. Whole brain grey matter voxel wise analysis

*Whole brain grey matter voxel wise analysis,displaying group effects of CBP vs. controls in brain responses to auditory (low/high) and pressure (low/high) stimulation.*

|  |  |  |  |  |  |
| --- | --- | --- | --- | --- | --- |
| **Region** | **Volume (mm^3^)** | **x** | **y** | **z** | **max/min Z** |
|  |  |  |  |  |  |
|  |  |  |  |  |  |
| **Low Intensity Auditory** |  |  |  |  |  |
| CBP > Control |  |  |  |  |  |
| Increases |  |  |  |  |  |
| R Brainstem | 96 | 3 | -41 | -62 | 3.52 |
| L Cerebellum | 216 | -29 | -60 | -29 | 3.37 |
| R Ctx_FEF | 528 | -18 | -38 | -16 | 3.90 |
| R Ctx_43 | 920 | 57 | -6 | 6 | 4.01 |
| L Ctx_A1 | 1488 | -45 | -22 | 8 | 4.03 |
| R Ctx_0P4 | 240 | 57 | -11 | 17 | 3.81 |
| R Ctx_A5 | 216 | 60 | 5 | -5 | 3.53 |
| R Ctx_6r | 352 | 57 | 13 | 0 | 3.47 |
| Hippocampus | 96 | -34 | -19 | -10 | 3.57 |
|  |  |  |  |  |  |
| Decreases |  |  |  |  |  |
| L Basal ganglia | 360 | -24 | -38 | 6 | -3.87 |
| R Cblm_VIIIb | 192 | 19 | -46 | -59 | -3.85 |
| L Cblm_VIIIa | 144 | -24 | -49 | -51 | -3.38 |
| L Cblm_IX | 144 | -5 | -52 | -46 | -3.52 |
| R Clbm_CrusI | 1240 | 49 | -68 | -40 | -4.08 |
| R Clbm_CrusII | 192 | 22 | -81 | -40 | -3.64 |
| L Clbm_VI | 216 | -29 | -38 | -35 | -3.46 |
| L Cblm_CrusI | 312 | -37 | -78 | -32 | -3.46 |
| L Cblm_V | 288 | 24 | -30 | -32 | -3.42 |
| R Cblm_CrusI | 456 | 22 | -76 | -29 | -3.79 |
| L Ctx_v23ab | 384 | -7 | -54 | 17 | -3.46 |
| R Ctx_RSC | 144 | 6 | -43 | 19 | -3.38 |
| R Ctx_RSC | 96 | 11 | -43 | -27 | -3.41 |
| R Ctx_PGs | 2480 | 46 | -68 | 38 | -4.29 |
| L Ctx_7m | 3040 | -5 | -60 | 35 | -4.96 |
| R Ctx_31pd | 792 | 14 | -57 | 38 | -4.65 |
| L Ctx_8Ad | 4160 | -26 | 26 | 49 | -4.43 |
| L Ctx_PGs | 2080 | -32 | -76 | 49 | -4.71 |
| R Ctx_8Ad | 480 | 27 | 34 | 49 | -3.50 |
| L Ctx_8Ad | 144 | -21 | 40 | 46 | -3.38 |
| L Ctx_STSvp | 336 | -50 | -35 | -8 | -3.93 |
| L Ctx_9p | 240 | -18 | 37 | 38 | -3.58 |
| L Ctx_PHA1 | 432 | -24 | -33 | -13 | -3.66 |
| R Ctx_PGp | 312 | 52 | -73 | 22 | -3.48 |
| R Ctx_PGi_R | 288 | 41 | -54 | 22 | -3.59 |
| L Ctx_TP0Js | 216 | -37 | -65 | 25 | -3.50 |
| R Ctx_IP1 | 168 | 30 | -76 | 46 | 3.74 |
| R Ctx_IP1 | 96 | 35 | -62 | 38 | -3.57 |
| R Ctx_5L | 96 | 14 | -49 | 65 | -3.40 |
| L Ctx_IFSa | 608 | -34 | 29 | 19 | -4.24 |
| L Ctx_a47r | 192 | -40 | 42 | -8 | -3.35 |
| L Ctx_a47r | 168 | -37 | 40 | -5 | -3.52 |
| L Ctx_p10p | 768 | -18 | 67 | 8 | -3.59 |
| R Ctx_p10p | 96 | 14 | 45 | -19 | -3.35 |
| L Ctx_13l | 216 | -18 | 24 | -16 | -3.65 |
| L Ctx_6mp | 288 | -7 | -19 | 73 | -3.61 |
| L Ctx_11l | 424 | -29 | 50 | -8 | -3.72 |
| CA1 Hippocampus | 936 | 41 | -25 | -13 | -4.32 |
| No label | 144 | -18 | 40 | -10 | -3.43 |
| No label | 192 | 49 | -46 | -8 | -3.52 |
| No label | 88 | -34 | 42 | -2 | -3.37 |
| No label | 144 | -7 | -14 | 35 | -3.65 |
|  |  |  |  |  |  |
|  |  |  |  |  |  |
| **High Intensity Auditory** |  |  |  |  |  |
| CBP > Controls |  |  |  |  |  |
| Increases |  |  |  |  |  |
| R Brainstem_Med | 216 | 6 | -35 | -46 | 3.36 |
| R Cblm_IX | 216 | 9 | -57 | -51 | 3.49 |
| L Cblm_VI | 584 | -26 | -57 | -29 | 3.74 |
| L Cblm_VI | 1224 | -13 | -65 | -16 | 3.88 |
| L Cblm_I_IV | 128 | -5 | -49 | 3 | 3.83 |
| R Ctx_FEF | 872 | 49 | -3 | 54 | 3.79 |
| R Ctx_43 | 672 | 57 | -6 | 6 | 3.69 |
| L Ctx_0P4 | 216 | -53 | -11 | 17 | 3.52 |
| L Ctx_6r | 360 | -53 | 10 | 3 | 3.68 |
| R Ctx_6r | 384 | 54 | 16 | 0 | 3.56 |
| Decreases |  |  |  |  |  |
| R Cau | 544 | 33 | -35 | 3 | -3.71 |
| R Cau | 288 | 19 | 24 | 17 | -3.60 |
| R Ctx_p32 | 288 | -17 | 53 | 3 | -3.44 |
| L Ctx_STSvp | 192 | -53 | -38 | -10 | -3.88 |
| R Ctx_MIP | 144 | 27 | -57 | 38 | -3.41 |
| L Ctx_PFt | 144 | -48 | -25 | 33 | -3.42 |
| R Ctx_5L | 312 | 14 | -41 | 60 | -3.46 |
| R Ctx_5L | 96 | 19 | -41 | 76 | -3.57 |
| L Ctx_8C | 288 | -32 | 18 | 38 | -3.69 |
| L Ctx_5m | 1320 | -2 | -35 | 60 | -4.29 |
| L Ctx_PF | 216 | -45 | -35 | 33 | -3.35 |
| CA1 Hippocampus | 192 | -32 | -41 | -2 | -3.59 |
| No label | 144 | 17 | -41 | 6 | -3.35 |
| No label | 144 | 27 | 13 | 41 | -3.44 |
| No label | 144 | 14 | 24 | 44 | -3.70 |
| **Low Intensity Pressure Pain** |  |  |  |  |  |
| CBP > Control |  |  |  |  |  |
| Increases |  |  |  |  |  |
| R Clbm_IX | 216 | 9 | -57 | -51 | 3.63 |
| R Ctx_44 | 288 | 52 | 16 | 6 | 3.63 |
| R Ctx_PH | 144 | 54 | -70 | -16 | 3.45 |
| L Ctx_6a | 384 | -26 | -17 | 54 | 3.55 |
| R Ctx_PF | 288 | 52 | -38 | 25 | 3.61 |
| R Ctx_24dv | 216 | 14 | -11 | 49 | 3.37 |
| L Ctx_AVI | 216 | -32 | 24 | -8 | 3.51 |
| Thal_LGN | 144 | 27 | -19 | -8 | 3.71 |
|  |  |  |  |  |  |
| Decreases |  |  |  |  |  |
| R Ctx_P0S2 | 288 | 14 | -60 | 38 | -3.39 |
| No label | 144 | 17 | -41 | 8 | -3.78 |
| **High Intensity Pressure Pain** |  |  |  |  |  |
| CBP > Control |  |  |  |  |  |
| Increases |  |  |  |  |  |
| L Cau | 144 | -26 | -33 | 0 | 3.66 |
| R Ctx_0FC | 360 | 6 | 13 | -24 | 3.90 |
|  |  |  |  |  |  |
| Decreases |  |  |  |  |  |
| L V_Striatum | 432 | -2 | 16 | -8 | -3.82 |
| L Cau | 384 | -24 | 26 | 3 | -3.69 |
| L Caudate_Cp | 528 | -7 | 18 | 11 | -4.17 |
| L Bstem_Midb | 144 | 1 | -25 | -13 | -3.35 |
| R Ctx_9m | 328 | 14 | 53 | 14 | 3.38 |
| R Ctx_9m | 96 | 11 | 56 | 19 | -3.34 |
| R Ctx_9a | 512 | 27 | 56 | 19 | -3.74 |
| L Ctx_TGd | 144 | -24 | 16 | -43 | -3.38 |
| R Ctx_MST | 384 | 41 | -62 | 8 | -3.73 |
| L Ctx_p10p | 552 | -26 | 48 | 3 | -3.87 |
| Haben | 144 | 3 | -22 | 6 | -3.48 |
| CA1_Hippocampus | 552 | 44 | -22 | -13 | -3.80 |
| No label | 336 | 30 | 48 | -2 | -3.81 |
| No label | 144 | 30 | -46 | 19 | -3.38 |

*Note*. Results are presented for the whole-brain grey-matter analysis at an exploratory threshold of p < .001 with 10 continuous voxels. Coordinates are in MNI space.

#### Table S6. Longitudinal Data availability

*Table showing how many participants in each study arm has usable data for pre- and post-intervention, pre-intervention only, or post-intervention only.*

|  | **Both Pre- and Post-Intervention** | **Pre-Intervention Only** | **Post-Intervention Only** |
| --- | --- | --- | --- |
| **PRT** | 42 | 7 | 1 |
| **Placebo** | 41 | 8 | 1 |
| **Usual Care** | 37 | 6 | 7 |

Comparing study arms, the PRT and placebo groups had similar rates of participants who had usable data at either the pre-intervention assessment only, the post-intervention assessment only, or at both assessments (Table 6, rows 1-2).

### 5. Supplementary Figures


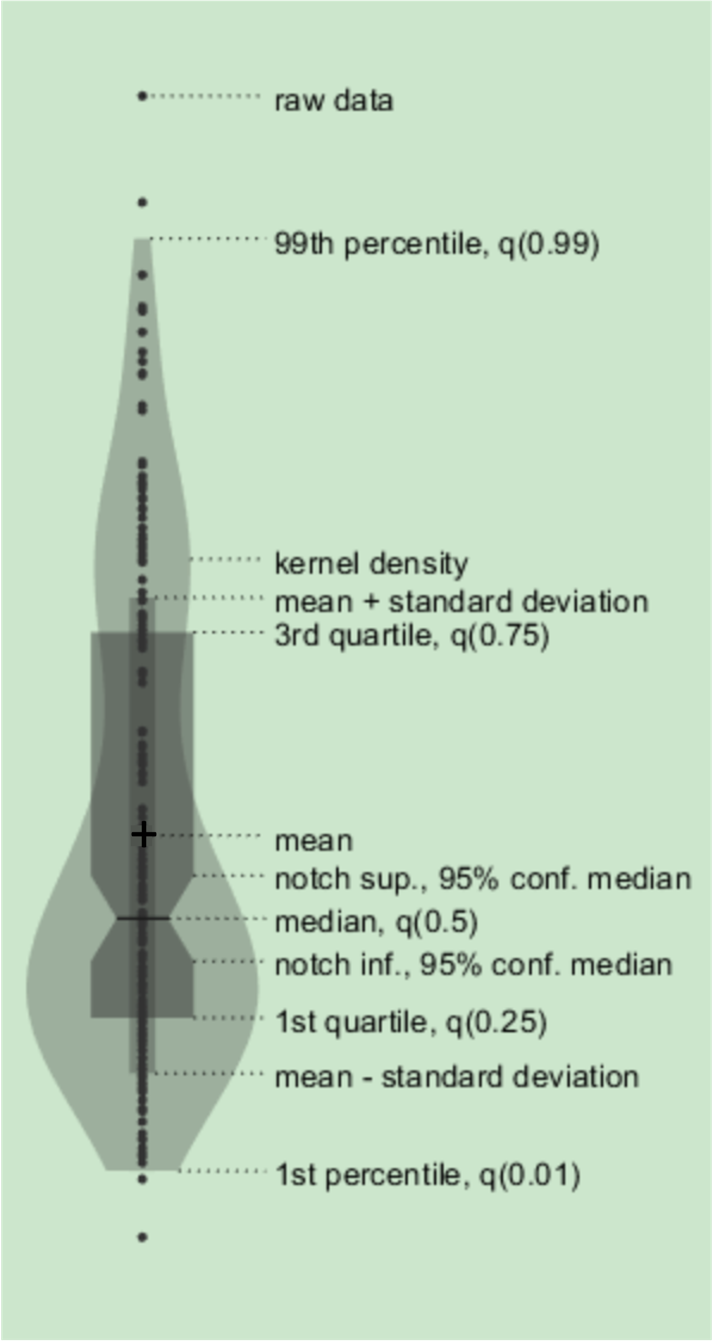


Figure S1. Detailed plot explanation. The plots display kernel density estimation with individual data points overlaid. Notched box plots within the violins show the median and interquartile range, while plus signs indicate the mean. The width of the violins reflects data density, and the spread illustrates response variability within each group.


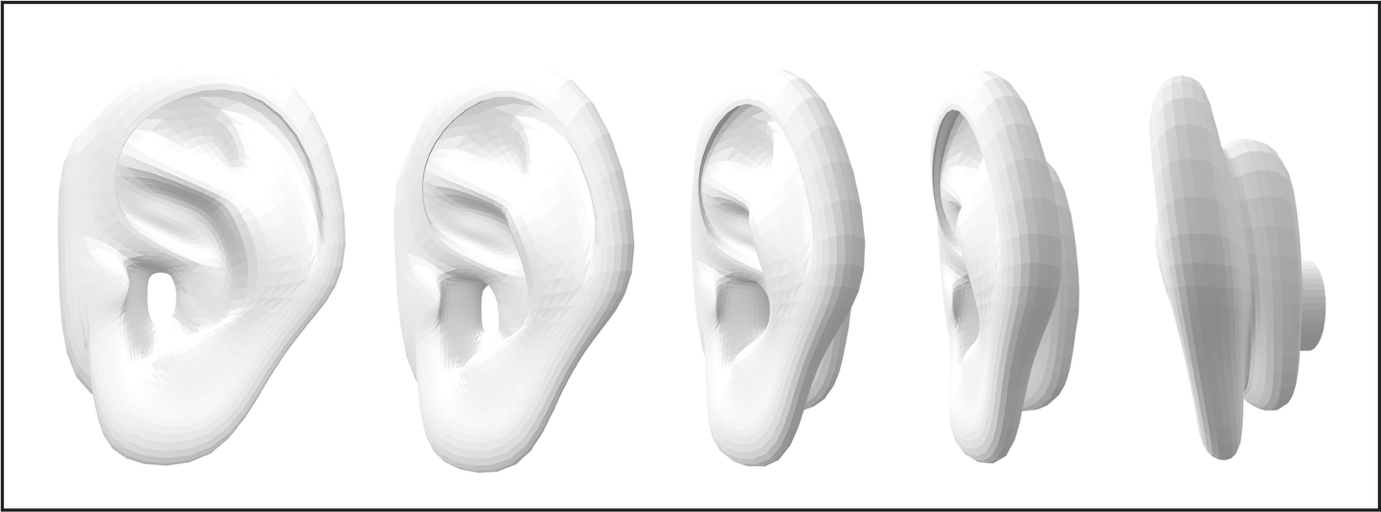


Figure S2. Artificial Ear Coupler Used to Verify Auditory Stimulus Intensities. 3D printed model used with calibrated decibel meter to confirm low- and high-intensity stimulus levels delivered via MRI-compatible earbuds.


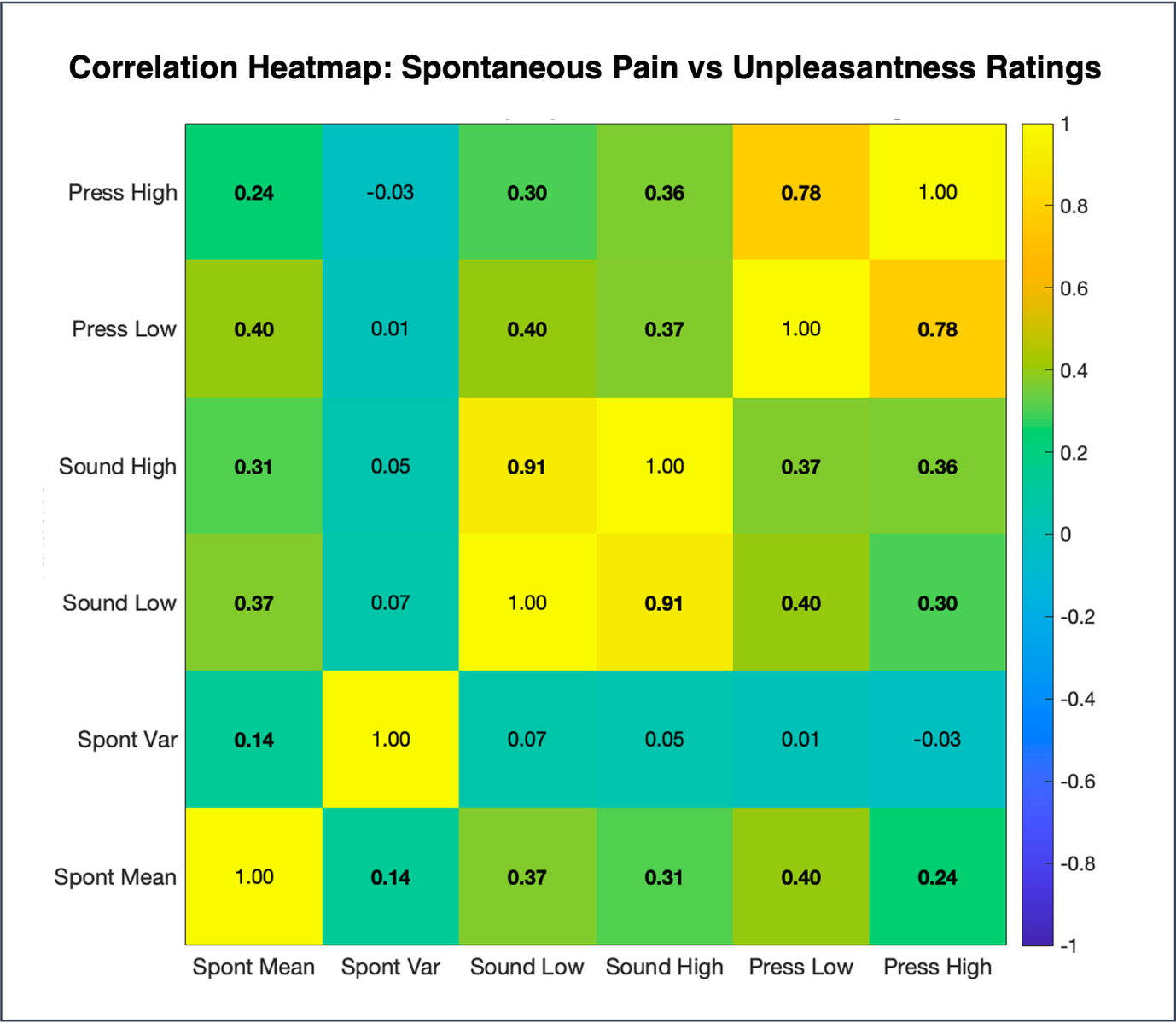


Figure S3. Correlation heatmap of spontaneous and task-evoked pain characteristics. Correlation heatmap showing associations between spontaneous pain characteristics (mean and variance during the resting scan) and task-evoked unpleasantness across the four stimulus conditions (sound low, sound high, pressure low, pressure high). Bold values indicate significant correlations (p < .05).


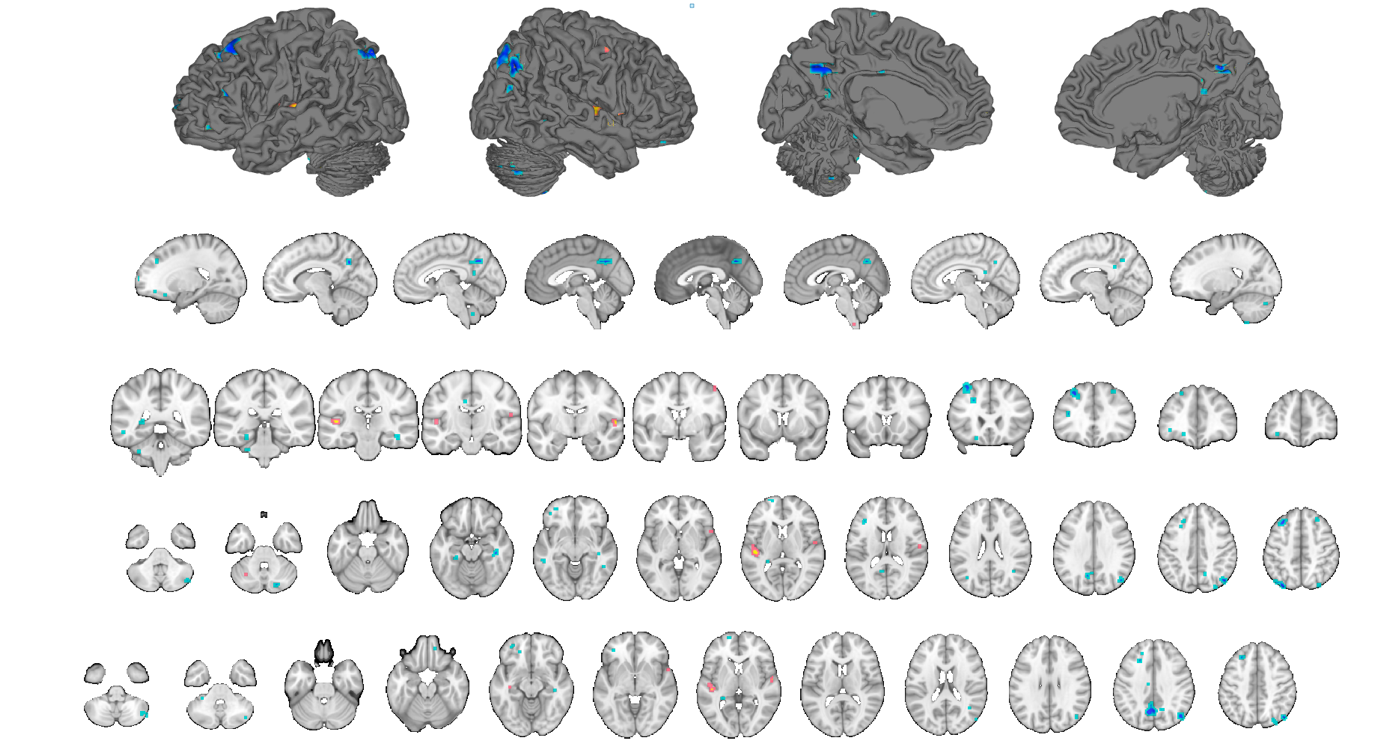


Figure S4. Low intensity auditory stimulation. Whole brain grey-matter analysis displaying differences in brain activity in people with CBP > controls in response to low intensity auditory stimulation. Clusters meet an exploratory threshold of *P* < 0.001 uncorrected. Yellow/orange areas display increased activity, whereas blue areas indicate hypoactivation in CBP vs controls.


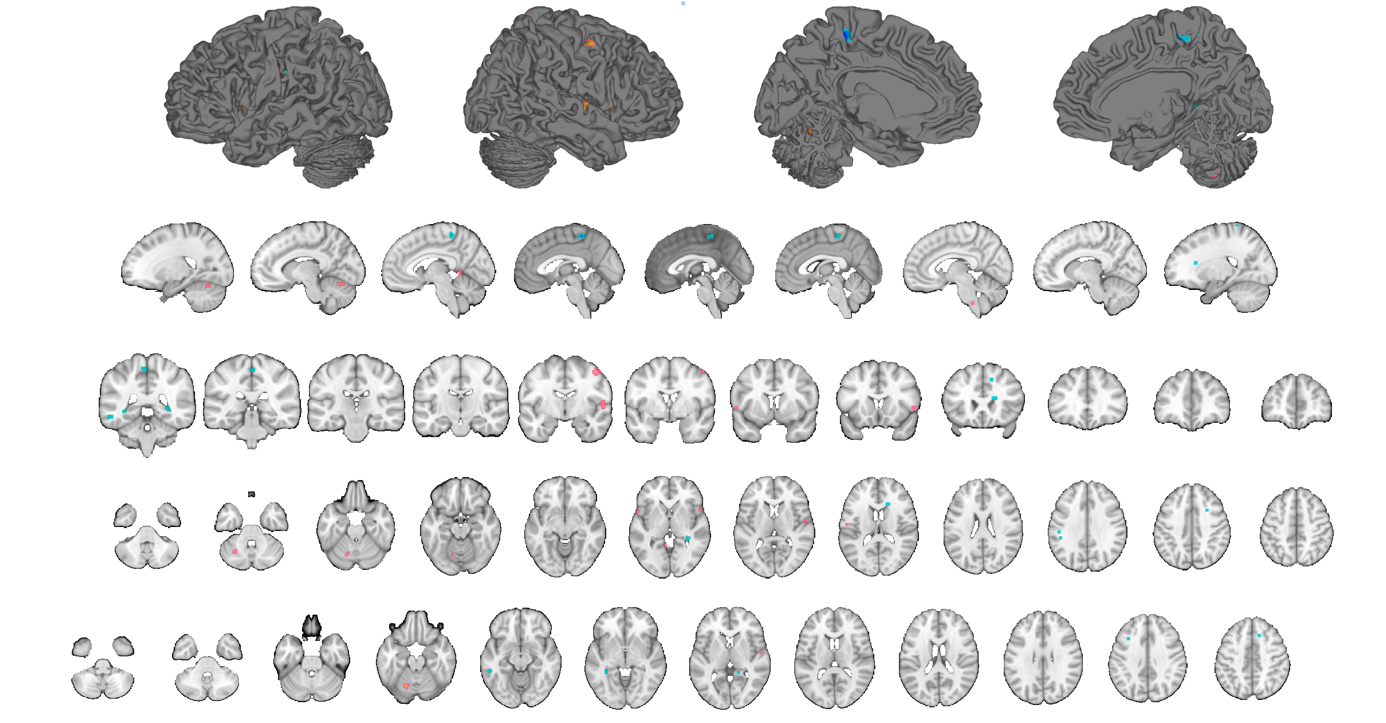


Figure S5. High intensity auditory stimulation. Whole brain grey-matter analysis displaying differences in brain activity in people with CBP > controls in response to high intensity auditory stimulation. Clusters meet an exploratory threshold of *P* < 0.001 uncorrected. Yellow/orange areas display increased activity, whereas blue areas indicate hypoactivation in CBP vs controls.


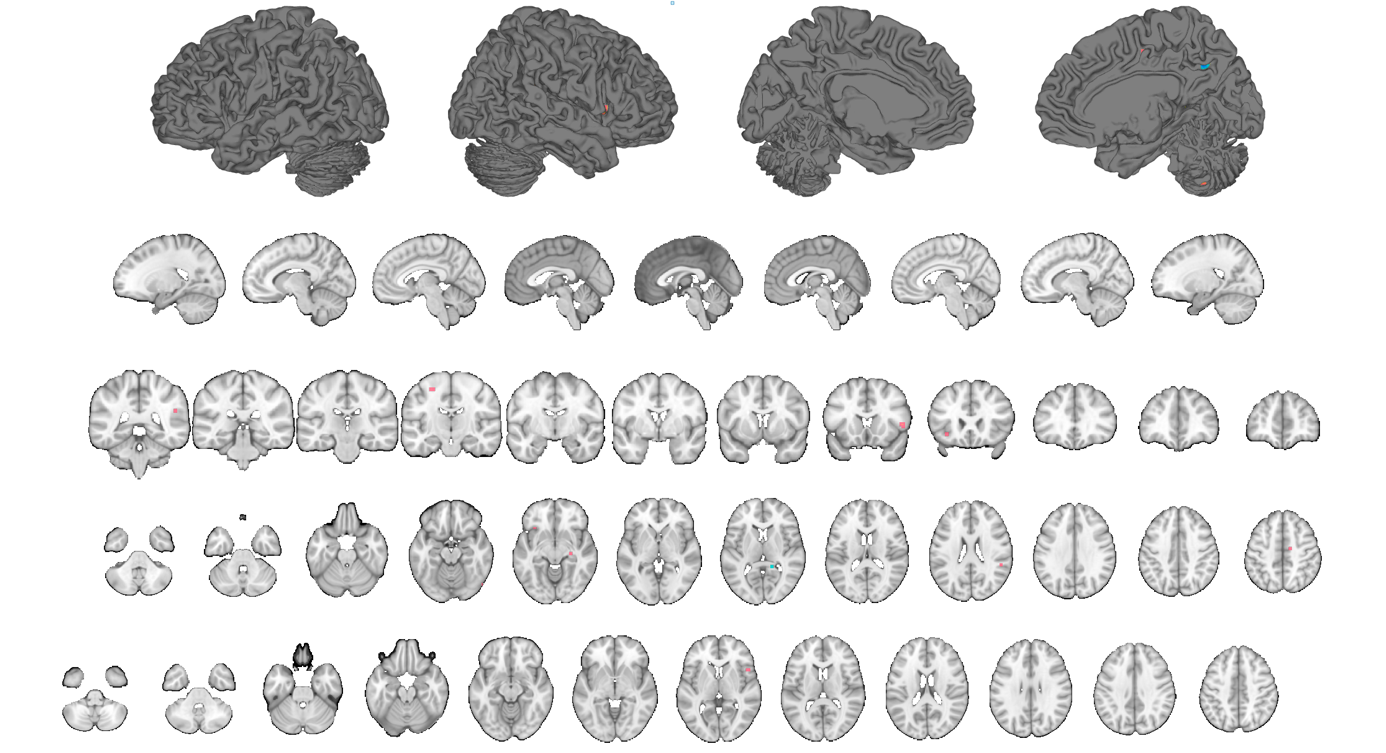


Figure S6. Low intensity pressure pain***.*** Whole brain grey-matter analysis displaying differences in brain activity in people with CBP > controls in response to low intensity pressure pain. Clusters meet an exploratory threshold of *P* < 0.001 uncorrected. Yellow/orange areas display increased activity, whereas blue areas indicate hypoactivation in CBP vs controls.


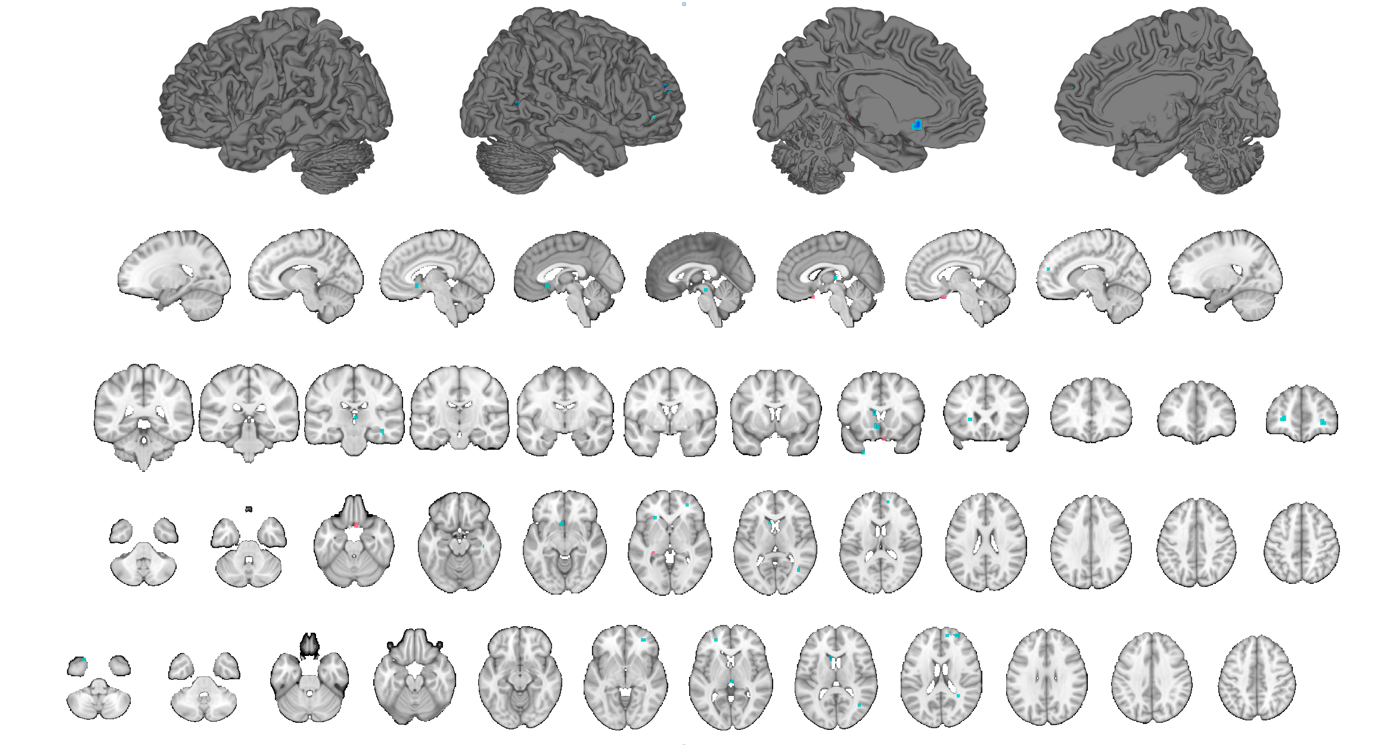


Figure S7. High intensity pressure pain***.*** Whole brain grey-matter analysis displaying differences in brain activity in people with CBP > controls in response to high intensity pressure pain. Clusters meet an exploratory threshold of P < 0.001 uncorrected. Yellow/orange areas display increased activity, whereas blue areas indicate hypoactivation in CBP vs controls.


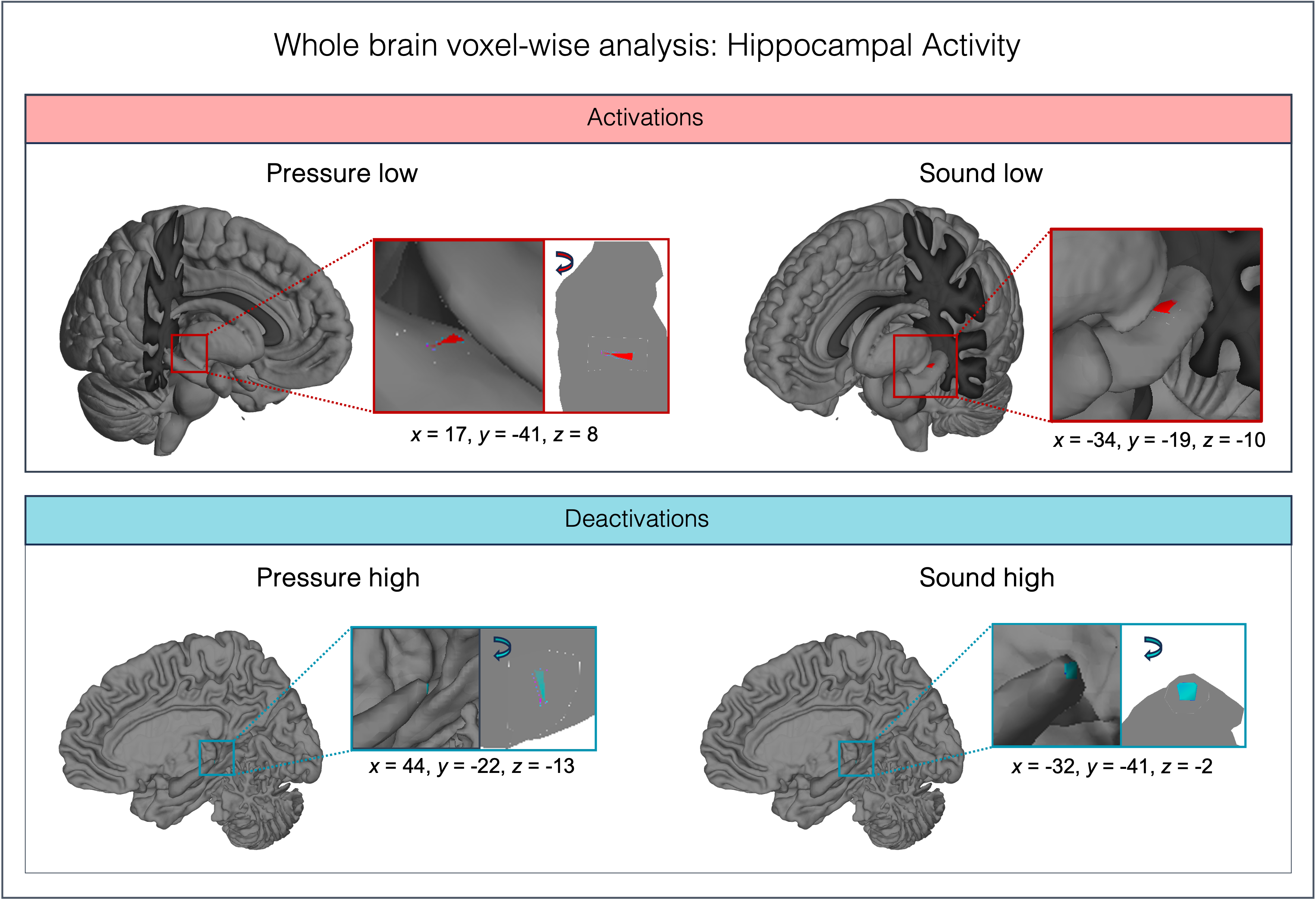


Figure S8. Whole-brain voxel-wise analysis: Hippocampal Activity. Differences in hippocampal activity between CBP patients and controls, with clusters at p < 0.001 uncorrected. Red areas show increased activation in CBP, while blue areas indicate reduced activation. Top: Activations during low-pressure and low-sound conditions. Bottom: Deactivations during high-pressure and high-sound conditions.

**
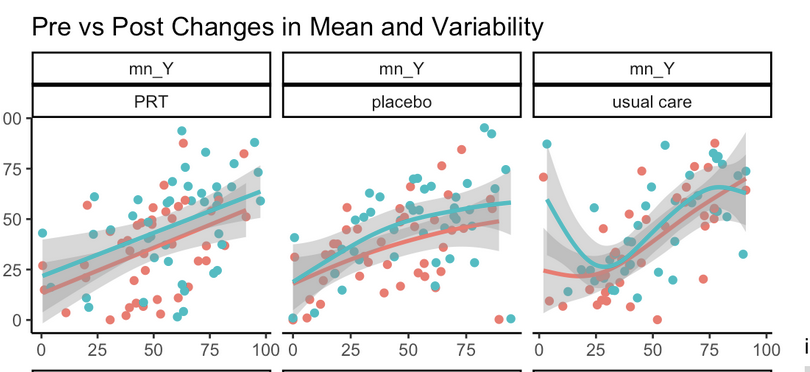
**

Figure S9. Scatterplot of average reactivity across sequential exposures in the pre-intervention assessment (x-axis) and post-intervention assessment (y-axis) separately by intensity (low=red, high=blue).

**
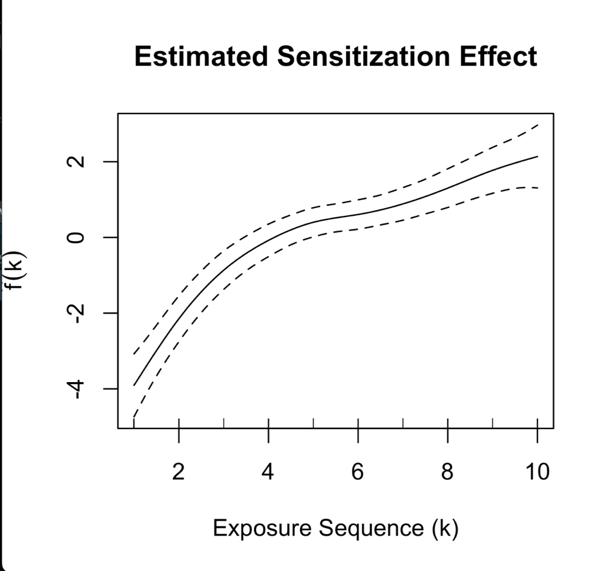
**

Figure S10. Estimated sensitization effect from the primary analysis. The estimated coefficient is plotted as a solid black line, with pointwise 95% confidence intervals indicated as dashed lines.

## 6. Discussion

Notably, whole-brain grey matter analysis revealed hippocampal CA1 deactivation in response to high intensity auditory and pressure stimuli in people with CBP compared to controls, while more anterior hippocampal activations were observed in the low sound and low pressure conditions (see Supplement Table 5, Figure S6). Given the established role of the hippocampal-mPFC pathway in spontaneous pain intensity ^16^, pain chronicity, and transition to chronic pain ^17^, and its involvement in memory ^18^ and anxiety ^19^, the observed MSS-related hippocampal activity changes in CBP may offer a promising avenue for future research.

Clinically, behavioral classifier outperformed the neural classifier, indicating that self-report measures may suffice for identifying patients with heightened sensitivity. Nonetheless, neuroimaging provides mechanistic insights into the pathways underlying sensory amplification.

## References

1. Esteban O, Markiewicz CJ, Blair RW, et al. fMRIPrep: a robust preprocessing pipeline for functional MRI. *Nat Methods*. 2019;16(1):111-116. doi:10.1038/s41592-018-0235-4

2. Gorgolewski K, Burns CD, Madison C, et al. Nipype: A Flexible, Lightweight and Extensible Neuroimaging Data Processing Framework in Python. *Front Neuroinformatics*. 2011;5. doi:10.3389/fninf.2011.00013

3. Tustison NJ, Avants BB, Cook PA, et al. N4ITK: Improved N3 Bias Correction. *IEEE Trans Med Imaging*. 2010;29(6):1310-1320. doi:10.1109/TMI.2010.2046908

4. Reuter M, Rosas HD, Fischl B. Highly accurate inverse consistent registration: A robust approach. *NeuroImage*. 2010;53(4):1181-1196. doi:10.1016/j.neuroimage.2010.07.020

5. Fonov V, Evans AC, Botteron K, Almli CR, McKinstry RC, Collins DL. Unbiased average age-appropriate atlases for pediatric studies. *NeuroImage*. 2011;54(1):313-327. doi:10.1016/j.neuroimage.2010.07.033

6. Cox RW, Hyde JS. Software tools for analysis and visualization of fMRI data. *NMR Biomed*. 1997;10(4-5):171-178. doi:10.1002/(SICI)1099-1492(199706/08)10:4/5%3C171::AID-NBM453%3E3.0.CO;2-L

7. Jenkinson M, Smith S. A global optimisation method for robust affine registration of brain images. *Med Image Anal*. 2001;5(2):143-156. doi:10.1016/S1361-8415(01)00036-6

8. Greve DN, Fischl B. Accurate and robust brain image alignment using boundary-based registration. *NeuroImage*. 2009;48(1):63-72. doi:10.1016/j.neuroimage.2009.06.060

9. Wager TD, Keller MC, Lacey SC, Jonides J. Increased sensitivity in neuroimaging analyses using robust regression. *NeuroImage*. 2005;26(1):99-113. doi:10.1016/j.neuroimage.2005.01.011

10. Lieberman MD, Cunningham WA. Type I and Type II error concerns in fMRI research: re-balancing the scale. *Soc Cogn Affect Neurosci*. 2009;4(4):423-428. doi:10.1093/scan/nsp052

11. Amunts K, Mohlberg H, Bludau S, Zilles K. Julich-Brain: A 3D probabilistic atlas of the human brain’s cytoarchitecture. *Science*. 2020;369(6506):988-992. doi:10.1126/science.abb4588

12. Kenward MG, Molenberghs G. Likelihood Based Frequentist Inference When Data Are Missing at Random. *Stat Sci*. 1998;13(3):236-247.

13. Wood SN. *Generalized Additive Models: An Introduction with R*. 2nd ed. Chapman and Hall/CRC; 2017. doi:10.1201/9781315370279

14. Team R. A language and environment for statistical computing. *Computing*. 2006;1. doi:10.1890/0012-9658(2002)083%5B3097:CFHIWS%5D2.0.CO;2

15. Wood SN, Pya N, Säfken B. Smoothing Parameter and Model Selection for General Smooth Models. *J Am Stat Assoc*. 2016;111(516):1548-1563. doi:10.1080/01621459.2016.1180986

16. Baliki MN, Chialvo DR, Geha PY, et al. Chronic Pain and the Emotional Brain: Specific Brain Activity Associated with Spontaneous Fluctuations of Intensity of Chronic Back Pain. *J Neurosci*. 2006;26(47):12165-12173. doi:10.1523/JNEUROSCI.3576-06.2006

17. Baliki MN, Petre B, Torbey S, et al. Corticostriatal functional connectivity predicts transition to chronic back pain. *Nat Neurosci*. 2012;15(8):1117-1119. doi:10.1038/nn.3153

18. Spellman T, Rigotti M, Ahmari SE, Fusi S, Gogos JA, Gordon JA. Hippocampal–prefrontal input supports spatial encoding in working memory. *Nature*. 2015;522(7556):309-314. doi:10.1038/nature14445

19. Adhikari A, Topiwala MA, Gordon JA. Synchronized Activity between the Ventral Hippocampus and the Medial Prefrontal Cortex during Anxiety. *Neuron*. 2010;65(2):257-269. doi:10.1016/j.neuron.2009.12.002
